# AI-Assisted Isolation of Bioactive Dipyrimicins from *Amycolatopsis azurea* and Identification of Their Corresponding Dip Biosynthetic Gene Cluster

**DOI:** 10.1021/acs.jnatprod.6c00057

**Published:** 2026-03-26

**Authors:** Christine Mae F. Ancajas, Isra Shuster, Allison S. Walker

**Affiliations:** † Department of Chemistry, 5718 Vanderbilt University, 1234 Stevenson Center Lane, Nashville, Tennessee 37240, United States; ‡ Department of Biological Sciences, Vanderbilt University, VU Station B, Box 35-1634, Nashville, Tennessee 37235, United States; § Department of Pathology, Microbiology, and Immunology, Vanderbilt University Medical Center, 1211 Medical Center Drive, Nashville, Tennessee 37232, United States

## Abstract

One of the major challenges in natural product discovery is the prioritization of compounds with useful activities from microbial sources. Here, we utilize a machine learning model that predicts the antibacterial activity of a natural product from its biosynthetic gene cluster (BGC) into our genome mining pipeline. Using this approach, we prioritized the strain *Amycolatopsis azurea* DSM 43854 as a candidate strain encoding multiple BGCs with antibacterial-producing potential. Through bioactivity-guided fractionation, dipyrimicins A and B were isolated and, for the first time, linked to their BGC. This *dip* BGC was predicted by our model to encode a product with 76% antibacterial probability and shares only 40–52% similarity with previously characterized BGCs. The antimicrobial properties of the dipyrimicins were confirmed against a few test strains, and putative tailoring enzymes were identified, including an O-methyltransferase and amidotransferase that differentiated them from other related 2,2′-bipyridine biosynthetic pathways. Importantly, as the *dip* BGC was not in the training set of the model, this demonstrates the ability of the model to generalize beyond its training set and the potential of machine learning to accelerate novel bioactive natural product discovery and deorphanization of BGCs.

## Graphical abstract

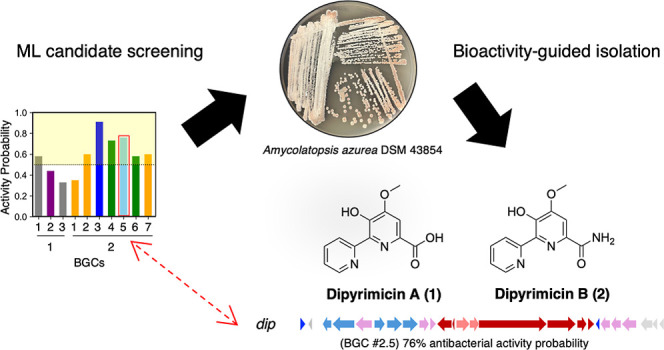

With the rise of antibiotic resistance, it has become increasingly important to find novel antibiotic compounds. Natural products, also known as secondary metabolites, are small molecules produced by bacteria, fungi, and plants and serve as a promising source of bioactive molecules. Many natural products have antimicrobial, antifungal, and cytotoxic properties, making them useful for the treatment of infectious disease and cancer. Currently, the majority of antibiotic compounds used therapeutically are natural products or natural product derivatives.^1^ As such, it is important to continue exploring the bioactivity of natural products because this can lead to the discovery of novel antibiotic compounds to combat the spread of antimicrobial-resistant pathogens.^2^

In bacteria, the genetic information for natural product biosynthesis is grouped together in what is known as the biosynthetic gene cluster (BGC), with each BGC encoding the enzymes responsible for natural product synthesis. While previous drug discovery efforts have showcased the bioactive potential of natural products, the classical methods used face recurring challenges such as the rediscovery of known compounds and difficulty prioritizing which BGCs are most promising.^3^ Although target-directed genome mining and recent computational tools have advanced BGC prioritization, many approaches rely on identifying conserved biosynthetic cores and resistance genes to infer potential bioactivity. Here, we apply a previously developed machine learning (ML) method that leverages genetic features across the BGC to estimate the likelihood that it encodes a natural product with antibacterial properties. Prior benchmarking of these models reported performance of up to 80% accuracy in cross-validation studies and up to 64% accuracy in held-out BGCs identified by antiSMASH and with no detected similarity to other clusters in the MIBiG database used to construct the training set.^4^ However, its implementation in guiding experimental discovery remains limited. We propose that integrating this ML-guided method, particularly when applied to rare actinomycetes and underexplored genus recognized as promising sources of antibiotics,^5^ can improve the efficiency of bioactive natural product discovery.

To test the accuracy of the ML model in tandem with experimental methods, we worked with *Amycolatopsis azurea* DSM 43854 as a promising candidate for antibiotic production. *A. azurea* DSM 43854 belongs to a rare genus of actinomycete with great potential for novel antibiotic discovery.^6^ Over 25 orphan BGCs with less than 75% similarity to characterized BGCs were predicted to produce an antibiotic compound. In this study, we used these predictions to guide our exploration of natural products produced by *A. azurea* DSM 43854. We isolated and purified antibiotic compounds produced by *A. azurea* DSM 43854, which we identified as dipyrimicins A and B. Dipyrimicins A and B were first identified and isolated from *Amycolatopsis* sp. K16-0194 in 2018 as antibiotic compounds, but the BGC of origin was unknown.^7^ Therefore, dipyrimicin BGC is not present in the training data used to train the machine learning model used in this study. Our work serves as a proof of concept for machine learning-guided discovery of antibiotic natural products, as the algorithm predicted that the BGC responsible for the production of dipyrimicins A and B would produce an antibiotic compound. Further in silico analysis identified the biosynthetic origin of dipyrimicins A and B, as well as specific enzymes likely responsible for the synthesis of specific functional groups within the compound.

## Results and Discussion

### Genome Mining and ML Model-Guided Screening Reveals Promising Novel BGCs

Secondary metabolites remain an important source of bioactive compounds. However, isolating active compounds is often hindered by the uncertainty of whether a given bacterial strain can produce metabolites with useful activities. To assess potential microbial sources for active compounds, specifically with antibiotic activity, we utilized a machine learning workflow, which uses annotations from antiSMASH 5 and RGI 5, to predict the antibiotic activity of BGCs within a given bacterial genome.^4,8,9^ We considered BGC activity prediction of 50% or greater as the “active” threshold as this represents a greater-than-even likelihood of producing an active compound. Based on these criteria, *A. azurea* DSM 43854 emerged as a promising candidate. First, antiSMASH analysis revealed that the genome of *A. azurea* DSM 43854 encodes 35 BGCs, many of which were <75% similar to known BGCs and classified across diverse natural product classes, including NRPS, PKS, RiPPs, terpenes, and more (Figure 1, Table S1). Moreover, of the 35 BGCs, 25 BGCs meet or exceed the “active” threshold for antibacterial activity (Figure 1, Table S2). To date, only two families of natural products have been isolated from *A. azurea*, whose associated BGCs remain putative. These are the antibiotic azureomycins A and B^10–12^ and antifungal octacosamicins A and B.^13,14^ The presence of several other orphan BGCs in *A. azurea* DSM 43854, such as clusters in contigs 2, 11, and 28, each with a high likelihood of producing diverse bioactive compounds (Figure 1), highlights the strain’s further untapped biosynthetic capacity and that it is an excellent starting point for our ML-prioritized discovery efforts.

**Figure 1:**
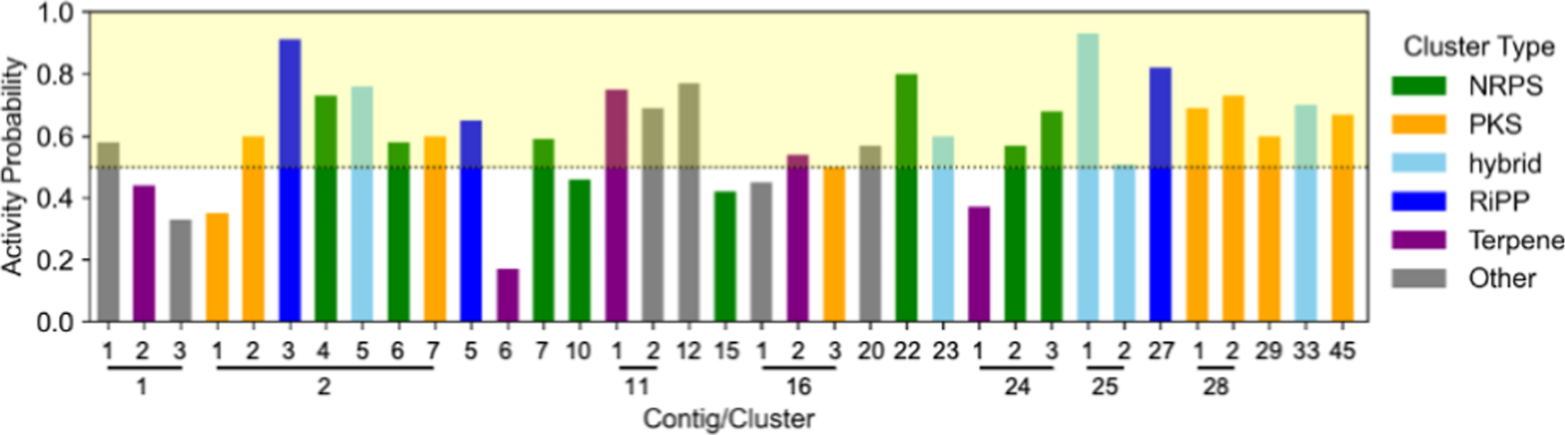
ML-based antibiotic activity prediction for each BGC in *Amycolatopsis azurea* DSM 43854. A probability threshold of ≥50% indicates higher antibiotic activity potential. Each BGC is color-coded by its antiSMASH predicted natural product class: green for NRPS, orange for PKS, light-blue for hybrid, dark-blue for RiPPs, purple for terpene, and gray for other. Clusters located on the same contig are grouped together, as indicated by the lines.

### Identification of Active Compounds

After identifying *A. azurea* DSM 43854 as a potential candidate for producing antibiotics, we proceeded to experimentally validate the BGC predictions by isolating bioactive metabolites. Initially, the strain was cultured under various media conditions, followed by liquid–liquid and solid-phase extractions. Crude extracts obtained were screened for antibacterial activity against *Bacillus spizizenii* ATCC 6633 by observing zones of inhibition. Extracts of cultures grown in ISP4 agar plates showed a notably higher zone of inhibition. We then proceeded to perform bioactivity-guided fractionation until individual active metabolites were isolated.

Of the fractions with notable antibiotic activity, two components—herein, compound 1, eluting first, and compound 2—exhibited prominent UV absorption spectra at around 205, 240, 270, and 320 nm (Figure S1), characteristic of aromatic chromophores. High-resolution ESI-MS analysis for 1 identified an *m*/*z* of 247.0708 [M + H]^+^ and *m*/*z* of 246.0871 [M + H]^+^ for 2 (Figure S2). To note, despite maintaining similar growth conditions, production of compound 2 was less reproducible across independent cultures, suggesting that its production may be influenced by subtle variations in growth conditions. The relative similarity in their retention time and UV absorption spectra suggest these are closely related compounds. Based on previous reports, these represent new metabolites isolated from *A. azurea* DSM 43854 as the observed *m*/*z* values differ from those of the known octacosamicins (*m*/*z* 625 and 639)^13,14^ and azureomycins (*m*/*z* > 850);^11^ to note, under our specific fermentation and extraction conditions, no *m*/*z* values corresponding to these two metabolites were detected.

To confirm the structures, comprehensive NMR experiments were performed. ^1^H NMR revealed aromatic signals (δ_H_ 7.40–8.59), correlating with the UV absorption data, and a methoxy group (around δ_H_ 4.05 and δ_C_ 56.51) in 1 and 2 (Figure 2, Table 1). Furthermore, detailed 2D NMR (COSY, HSQC, and HMBC) analysis confirmed the aromatic presence as a 2,2′-bipyridine (2,2′-BP) core (Figures S3–S12), which aided in our analysis. Database and literature search identified similarities with the well-characterized 2,2′-BP-containing natural products collismycins and caerulomycins.^15^ Additionally, the reported NMR by Izuta et al.^7^ aligns with our observed connectivities of the hydroxyl group on C3′, methoxy group on C4′, aromatic proton H5′, and carbonyl group on C7′ on the more substituted ring. The 1 *m*/*z* difference between compounds 1 and 2 can be attributed to presence of the hydroxyl or amine functional group on C7′, respectively (Figure 2). This is in agreement with the chemical formulas C_12_H_10_N_2_O_4_ (calcd. 247.0719) and C_12_H_11_N_3_O_3_ (calcd. 246.0879) as well as the observed *m*/*z* values reported both in this study (Figure S2) and by Izuta et al.^7^ Therefore, in accordance with the previous data, compounds 1 and 2 from the active fraction are, hereafter, confirmed as dipyrimicin A and dipyrimicin B, respectively.

**Figure 2:**
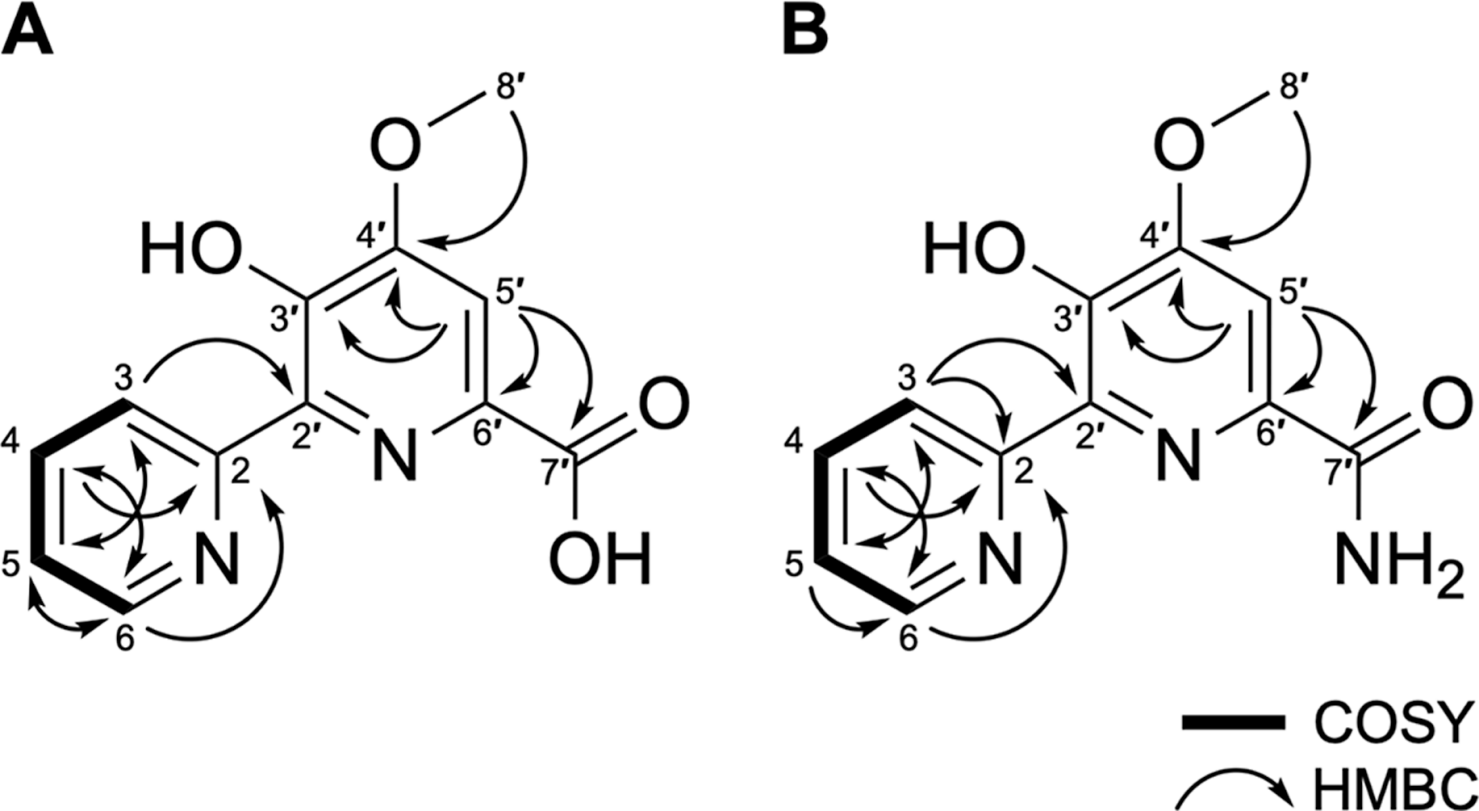
Key COSY and HMBC correlation observed for (A) dipyrimicin A (compound 1) and (B) dipyrimicin B (compound 2) isolated from *A. azurea* DSM 43854.

**Table 1:** ^1^H (600 MHz) and ^13^C (150 MHz) NMR Data of Dipyrimicin A (1) and Dipyrimicin B (2) in CDCl_3_

	Dipyrimicin A (1)		Dipyrimicin B (2)	
position	δ_C_, type	δ_H_ mult. (*J* in Hz)	δ_C_, type	δ_H_ mult. (*J* in Hz)
2	156.55		157.62	
3	120.91	8.50, d (8.1)	120.93	8.54, m
4	138.66	8.03, td (7.8, 1.8)	138.25	7.95, td (7.8, 1.8)
5	124.03	7.47, ddd (7.5, 5.0, 1.1)	123.50	7.40, ddd (7.5, 5.0, 1.2)
6	145.62	8.59, dd (4.5, 1.4)	145.55	8.55, m
2′	133.94		134.08	
3′	151.15		149.84	
4′	156.90		156.16	
5′	107.06	7.76, s	106.39	7.79, s
6′	137.33		140.83	
7′	164.46		167.17	
8′	56.72	4.06, s	56.51	4.04, s
3′–OH				
7′–OH		-		
7′-NH2				-

### Bioinformatic Analysis of Dipyrimicin BGC and Biosynthesis

Having confirmed the production and initial activity of dipyrimicins A (1) and B (2) by *A. azurea* DSM 43854, we analyzed the genome to link the isolated compounds to their biosynthetic origin. Dipyrimicins, first reported by Izuta et al. in 2018, were originally isolated from a closely related strain, *Amycolatopsis* sp. K16-0194.^7^ Since then, dipyrimicin has also been reported from *Streptomyces thioluteus*,^16^ along with other 2,2′-BP analogs from various strains.^17^ However, high-quality genome assemblies and BGCs responsible for these compounds have yet to be reported. Moreover, identification of this uncharacterized BGC is of particular interest, as it was absent in the training data used to develop the ML model applied in this study, which allows us to evaluate whether the model can generalize beyond its training set. Here, to identify the dipyrimicin BGC (*dip*), we refined our search to the 25 BGCs with high activity probabilities. Among these, we identified cluster 2.5 (76% antibacterial activity probability) as a putative *dip* BGC (Figure 1). This cluster shares 52% and 40% similarity with the BGCs for caerulomycin A (*cae*) and collismycin A (*col*), respectively.

As the dipyrimicins share the 2,2′-BP core of the caerulomycins and collismycins, we examined the putative *dip* BGC for the presence of genes conserved in their respective pathways. Comparison of the annotated genes within the putative *dip* BGC to those of *cae* and *col* BGCs indicated that all of the genes necessary for the formation of a 2,2′-BP core were present, ranging from 25% to 79% identity (Table S3). Specifically, *dip11–17* shows close homology to the conserved *cae/col A1-A4*, *P1–P2*, and *B1* genes, which form the 2,2′-bipyridine- *l* -leucine intermediate as well as the transporter genes *cae/col H1–H2* (Figure 3A).^18,19^ Distant homologues of the pathway-specific regulators, *cae/colI1-I2*, were also present.^20^ However, the *dip* BGC lack the genes (*cae/col B2*, *F*, *C*, and *caeB5*) likely involved in forming the oxime group characteristic of the caerulomycins and collismycins (Table S3).^21–24^ Subsequently, we identified notable *dip* genes, including those predicted to encode an O-methyltransferase and amidotransferase, which differ from either of the known *cae* and *col* pathways. These enzymes are hypothesized to decorate the 2,2′-BP core to afford dipyrimicins (Figure 3B). While the functional groups installed are not entirely unique to the dipyrimicins, identifying the enzymes responsible for these functionalities supports the assignment of the *dip* BGC and provides new insights into the biosynthetic pathways within this family of compounds.

**Figure 3:**
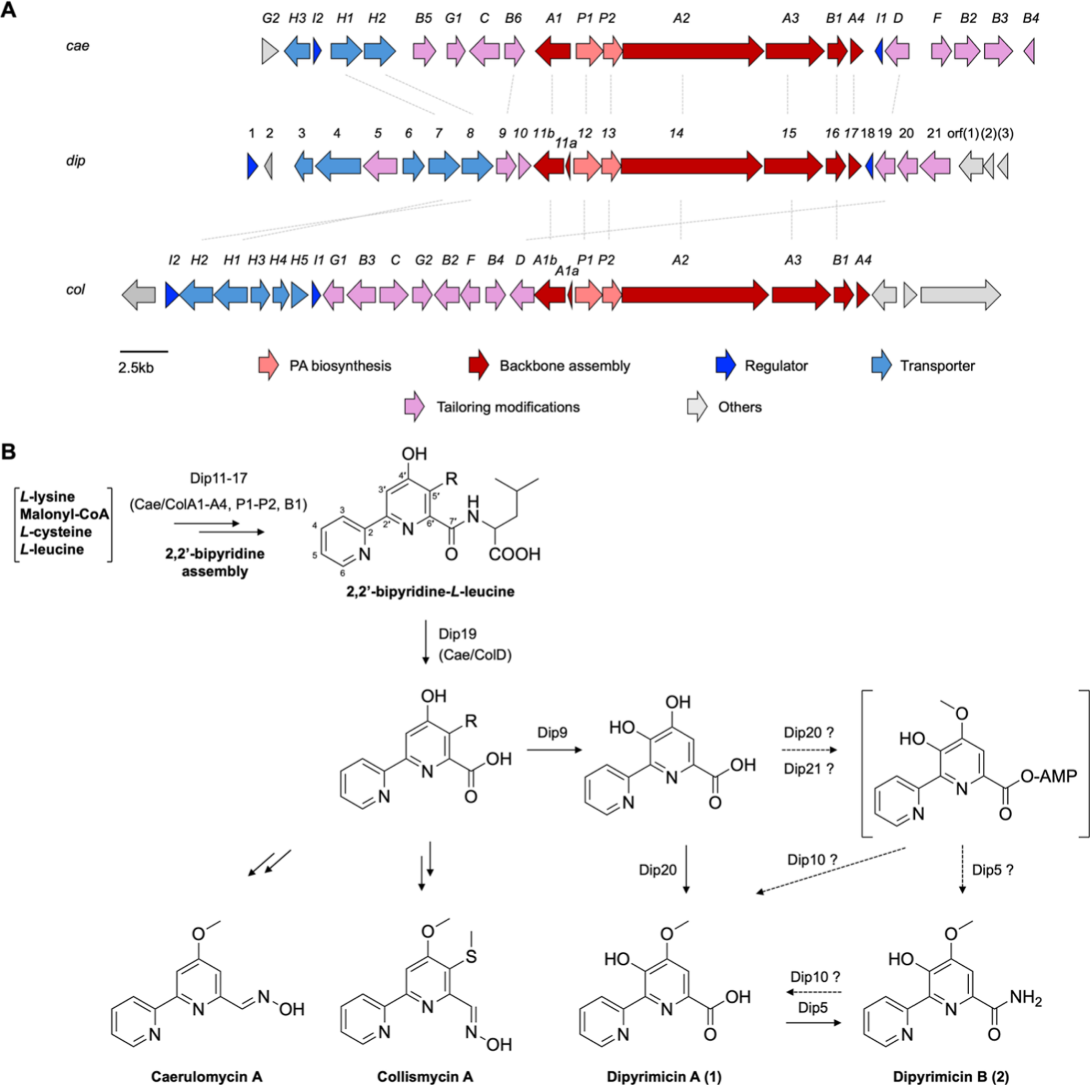
Proposed biogenesis of 2,2′-BP natural products. (A) Alignment of the caerulomycin A BGC (*cae*) from *Actinoalloteichus* sp. WH1-2216-6, putative dipyrimicin A (1) and B (2) BGC (*dip*) from *A. azurea* DSM 43854, and collismycin A BGC (*col*) from *Streptomyces* sp. CS40. Alignments with >50% identity are linked by dashed lines. Standard nomenclature for *cae* and *col* by the Liu group was adapted.^25^ (B) The proposed biosynthesis of dipyrimicins follows a similar pathway to caerulomycins and collismycins through the formation of a 2,2′-bipyridine (2,2′-BP) scaffold. Variation in tailoring modifications differentiate them, such as the oxime group in caerulomycin A and collismycin A. Based on sequence and structural homology, with high confidence, dipyrimicins A and B are putatively hydroxylated at C3′ and O-methylated at C4′, while dipyrimicin B is further amidated at C7′ by Dip9, Dip20, and Dip5, respectively. Though the predicted function of Dip10 and Dip21 has low confidence, an alternative pathway in which Dip21-Dip20, similar to hybrid AMP-ligase and methylase QbsL, may C4′-methylate and activate the carboxylic acid to form the acyl-adenylate intermediate. Dip5 and putative Dip10 hydrolase may function to form dipyrimicin B and regenerate the carboxylic acid in dipyrimicin A.

First, the presence of *dip19*, which is predicted to encode an amidohydrolase with 73% and 61% identities to CaeD (83% similarity) and ColD (73% similarity), respectively, suggests that Dip19 catalyzes the same hydrolysis reaction, converting the 2,2′-BP- *l* -leucine intermediate into the carboxylated 2,2′-BP retained in dipyrimicin A (Figure 3B).^26^ The other tailoring gene with relative homology within the *cae* BGC is *dip9*, which is predicted to encode a NAD­(P)/FAD-dependent oxidoreductase (65% identity, 71% similarity) and shows the highest level of sequence homology to *caeB6* (54% identity, 66% similarity). In caerulomycins, CaeB6 has been experimentally implicated to catalyze the oxygenation at C3′.^27^ Therefore, it is likely that Dip9 putatively hydroxylates C3′ in the dipyrimicins, as well. To note, no homology of this gene is found in the *col* BGC and, as expected, there are no reported C3′-hydroxylated collismycins.

Similar to several caerulomycins and collismycins, dipyrimicins are O-methylated at C4′. In the *cae* and *col* pathways, this formation is processed by O-methyltransferases CaeG1 and ColG1, respectively.^21,28^ Previous experimental studies by Chen et al. on the *cae* pathway revealed a competition between the CaeB6 C3′-hydroxylation and CaeG1 C4′–OH methylation of caerulomycin H, an oxime-containing C4′-O-demethylated 2,2′-BP intermediate. The main route leading to caerulomycin A involves direct C4′–OH methylation, where C3′ remains unmodified. In the minor route, CaeB6 hydroxylates C3′ before CaeG1 C4′–OH methylation.^27^ Within the putative *dip* BGC, a hypothetical protein, Dip20, shares the highest similarity to SAM-dependent methyltransferases (65% identity and 74% similarity) but exhibits low homology to CaeG1 (25% identity and 42% similarity) and ColG1 (26% identity and 44% similarity). BlastP analysis from MIBiG reveals that Dip20 has close homology to QbsL (43% identity, 63% similarity) in the siderophore quinolobactin pathway.^29^ QbsL is a hybrid protein with an AMP-dependent ligase and synthetase domain (N-terminal) and a methylase domain (C-terminal), facilitating the carboxylic acid activation and O-methylation of a hydroxyl group, respectively, on a precursor xanthurenic acid.^30^ We propose that the dipyrimicin pathway adopts the minor route in caerulomycin biosynthesis as its primary pathway, where Dip9 hydroxylates C3′, followed by C4′–OH methylation by Dip20. The resulting formation of the C4′-methoxy group likely serves as a protecting group, preventing additional modifications of the hydroxyl group that could potentially lead to 2,2′-BP glycosylated cyanogrisides and other derivatives.

The amido group in dipyrimicin B (2) has been observed in a few 2,2′-BP metabolites as synthetic derivatives (e.g., caerulomycinamide),^16,31,32^ shunt products (e.g., collismycin DS),^22^ and/or isolated from bacterial cultures (e.g., caerulomycinamide and saccharobipyrimicin 1),^16,17^ yet no specific enzyme has been linked to its formation. To investigate a possible enzyme for this modification, we examined *dip5*, which encodes a putative amidotransferase with high similarity to glutamine-dependent asparagine synthetases (62% identity, 73% similarity) known to catalyze the ATP-dependent transfer of a glutamine-derived amino group to aspartate.^33^ Additionally, Dip5 shows homology to DacD (51% similarity, 63% identity) and other N-terminal-nucleophilic (Ntn) enzymes involved in conversion of a malonate-equivalent starter unit to a corresponding malonamate in tetracycline family biosynthesis.^34,35^ Based on these similarities, Dip5 may also catalyze an amine transfer at C7′, converting dipyrimicin A into amidated dipyrimicin B.

While the putative tailoring genes encoding enzymes that modify the 2,2′-BP core to produce the dipyrimicins have been annotated through sequence analysis, the availability of advanced structure prediction technologies, such as AlphaFold3, enables the integration of sequence and structural data to further refine the functional assignments of these tailoring enzymes. Using the implementation of AlphaFold3 in AlphaFold Server,^36^ protein models for these enzymes were generated, and FoldSeek^37^ was used to search the structural space within the Protein Data Bank (PDB) and AlphaFold Database (AFDB). For example, since Dip9 shows high sequence homology to Cae/ColB6, it is no surprise that proteins with similar structures identified by FoldSeek belong to the oxidoreductase superfamily, although the highest sequence identities are only around 36–39% (Table S4). Also, the AlphaFold-predicted structure of Dip9 showed strong alignment to the predicted model of CaeB6 and other related hydroxylases (Figure S13). Similarly, structural analyses of the Dip20 O-methyltransferase, along with the predicted model of QbsL, confirmed its classification within the SAM methyltransferase superfamily (Table S5), as indicated by the conserved “DxGxGxG” motif for SAM binding (Figure S14).^38^ For the putative Dip5 amidotransferase, the closest structural homologues in the AFDB were glutamine-dependent asparagine synthetases, with sequence identity as high as ∼45% (Table S6). Structural alignment with related proteins, including the predicted structure of DacD, confirms that Dip5 contains the conserved ATP-binding motif (“SGGLDS”)^39^ and the distinct N-terminal glutaminase and C-terminal synthase domains (Figure S15). Furthermore, similar to the hydroxyamidotransferase TsnB9 (PDB 7YLZ), Dip5 possesses inserted regions that differentiate it from the canonical asparagine synthetase, AsnB from *Escherichia coli* (PDB 1CT9), potentially allowing it to accommodate the dipyrimicin acid substrate (Figure S15).^40^

Lastly, the potential biosynthetic roles of two genes, *dip10* and *dip21*, remain unaccounted for. Dip21 is located adjacent to the putative Dip20 O-methyltransferase and is predicted to function as an AMP-dependent synthetase and ligase (Table S3), sharing 48% identity and 64% similarity with QbsL. Notably, the xanthurenic acid processed by QbsL methylates a hydroxyl group meta to a carboxylic acid activated by the ligase, a similar structural arrangement observed in the dipyrimicin acid.^30^ Structural comparisons indicate that the separate AlphaFold models of Dip20 and Dip21 align well with the C-terminal methylase and N-terminal synthetase domains of the AlphaFold QbsL structure, respectively, suggesting similar functions (Figure S16). FoldSeek search revealed homologues with ligase activity including aminoacyl-AMP synthetase and CoA ligases (Table S7). While the strong sequence and structural similarity of Dip5, Dip9, and Dip20 to enzymes within characterized pathways support their classification as high-confidence contributors to dipyrimicin assembly, the roles of Dip10 and Dip21 remain highly speculative. If Dip21 is functional, its role as an activator may be relevant as the intermediate it could form, such as dipyrimicin-AMP, would be highly susceptible to nucleophilic attack. In the quinolobactin biosynthesis, QbsL-mediated carboxyl activation facilitates the subsequent sulfurylation and reduction by QbsCDEK, leading to the formation of a thioacid before a proposed spontaneous hydrolysis regenerates the carboxyl group.^41^ However, homologues of these sulfurylation and reduction enzymes are absent in the *dip* BGC. Furthermore, while the acyl-adenylate intermediate formed could theoretically be targeted by an N-source for amidation, Dip5 has been shown to contain the corresponding domains to catalyze its own adenylation (Figure S15), which is typical for most asparagine synthetases,^33^ suggesting a different purpose for Dip21 activation. Meanwhile, Dip10 exhibits the highest sequence similarity to an α/β-hydrolase (67% identity and 76% similarity). Homology searches against MIBiG reveal similarities to the Ntn hydrolase PvdQ from the pyoverdine siderophore biosynthetic pathway (45% similarity, 61% identity), which catalyzes the hydrolysis of a pyoverdine precursor and a fatty acid chain.^42^ Structural homology hits from FoldSeek identified related hydrolases (Table S8). If Dip10 functions analogously to these related proteins, it may hydrolyze at the electrophilic center of an intermediate to regenerate the carboxyl group in dipyrimicin A.

Based on sequence and structural analyses, we propose the biosynthetic pathway of dipyrimicins in *A. azurea* DSM 43854 (Figure 3B). Our hypothesized pathway follows the conserved 2,2′-BP formation but diverges from the known caerulomycin and collismycin pathways after Dip9-mediated C3′-hydroxylation. Instead, dipyrimicin biosynthesis appears to undergo a distinct O-methylation mechanism in which Dip20 O-methyltransferase is adjacent to a Dip21 AMP-ligase. However, whether Dip20 and Dip21 function cooperatively, as in the bifunctional QbsL, remains uncertain. The low sequence and structural homology of Dip20 to Cae/ColG1 may not only reflect differences in substrate specificity or enzymatic promiscuity but also indicate that Dip20 was acquired from a distinct yet related siderophore biosynthetic pathway. Unlike Dip5, Dip9, and Dip20, where the corresponding enzymatic reactions and modifications in the dipyrimicin biosynthesis are well-supported, the roles of Dip21 and Dip10 remain uncertain, as the pathway could be complete without them. To complete the *dip* BGC annotation, *dip3*, *dip4*, and *dip6* are predicted to encode ABC and MFS transporters likely involved in the export of the natural product while *dip2* encodes a hypothetical protein with unknown function (Table S3). To validate our bioinformatic analyses, experimental studies will be essential to confirm the functional roles especially for the post-tailoring modifying enzymes encoded within the putative *dip* BGC. In addition, further experiments on clusters with different tailoring enzymes knocked out could provide useful training data for the model. If a tailoring enzyme knockout resulted in an alternative product without activity, then incorporation of the BGC lacking the tailoring enzyme into our training set would enable our model to better learn that the tailoring enzyme installed a modification essential for activity in that compound subfamily.

### Biological Activity

Through bioactivity-guided fractionation and the identification of *dip* BGC, we confirmed that the ML-predicted BGC produces bioactive metabolites. However, our ML prediction reflects the confidence in antibacterial activity but does not quantify potency. Therefore, we further characterize the antimicrobial activity of the dipyrimicins by determining their minimum inhibitory concentrations (MICs) and establishing preliminary structure–activity relationship (SAR) trends.

Previous susceptibility studies using the disk diffusion method reported moderate antimicrobial activity of dipyrimicin A with growth inhibitions of 16–23 mm at doses of 30 and 100 μg dose against *E. coli* NIHJ and *B. spizizenii* ATCC 6633. In contrast, dipyrimicin B only exhibited activity at 100 μg against *E. coli* NIHJ.^7^ Here, we confirm the minimum inhibitory concentration (MIC) of the dipyrimicins via broth microdilution. Dipyrimicin A displayed moderate activity against Gram-positive *B. spizizenii* ATCC 6633 (32 μg/mL) and *Staphylococcus aureus* ATCC 25923 (64 μg/mL), while its activity was reduced against Gram-negative *E. coli* MG1655 ATCC 700926 (128 μg/mL) and *Pseudomonas aeruginosa* (>128 μg/mL). Consistent with previous bioassays, dipyrimicin B displayed weaker activity with partial inhibition at 128 μg/mL for both *B. spizizenii* ATCC 6633 and *E. coli* MG1655 ATCC 700926 (Table S9). These findings suggest that the Dip5 amidation on C7′ in dipyrimicin B diminishes antibacterial activity. Moreover, these MIC values are relatively weak in comparison to those reported for other 2,2′-BP natural products, which have demonstrated activity as low as 2.5 to 5 μg/mL against *E. coli* and *P. aeruginosa*, respectively, by caerulomycin A,^31^ and 8 μg/mL against methicillin-resistant *S. aureus* (MRSA) by collismycin A.^43^ This discrepancy in antibacterial activity highlights the well-established importance of the oxime group in enhancing the antibacterial activity of the 2,2′-BP compounds.

However, the presence of the carboxylic and amido group may serve other biological roles beyond antibacterial activity. For example, previous studies on caerulomycin A analogs revealed that conversion of the aldoxime to a carboxylic or amido functional group preserved their phytotoxic properties. This was attributed to their ability to form complexes with iron.^44^ It is well-documented that 2,2′-BP compounds have iron-chelating properties and have been implicated in their diverse bioactivities.^15^ For example, collismycin C, originally reported to exhibit poor antibacterial activity,^45^ was later identified as a potent inhibitor of MRSA biofilm formation, which is a property speculated to arise from its iron-chelating ability and the positioning of the hydroxyl group on the 2,2′-BP core.^46^ More recently, Henriquez et al. proposed that these metal-chelating compounds of the 2,2′-BP family may disrupt intracellular ion homeostasis, leading to microbial metabolic stress and eventual growth inhibition.^47^ Thus, further studies could explore additional bioassays to better understand the mechanism of action of dipyrimicins.

In addition to these SAR considerations, the disparity between the high ML prediction score for the *dip* BGC and the modest MIC values observed experimentally highlights the weakness of the current ML model. The model is trained to estimate the likelihood of activity but not predict level of activity or mechanism. Furthermore, the training data set contains a substantial portion of Gram-positive activity measurements, many against *Bacillus subtilis*, which may bias prediction toward Gram-positive activity rather than broad-spectrum or clinically relevant targets.^4^ More accurate prediction of activity against clinically important pathogens would require retraining on pathogen-specific data sets, including ESKAPE species, with potency measurements, which are currently not widely available across natural products.

### Expanding Targeted Genome Mining with ML

With the successful identification of a bioactive natural product and its previously unreported BGC, we next explored how this approach could be extended to systematically mine other 2,2′-BP-producing strains. By integrating ML-based activity predictions with targeted genome mining of conserved 2,2′-BP core biosynthetic genes, we identified several promising BGCs with ≥50% activity probability (Table S10) in previously unreported strains such as *Nocardia panacis*, *Micromonospora craniellae*, and *Micromonospora tulbaghiae* as well as other species within the *Amycolatopsis* and *Streptomyces* genera. Interestingly, we also identified *Streptomyces caatingaensis*, containing the same gene arrangement as the putative *dip* BGC in *A. azurea* DSM 43854, with >50% sequence identity (Figure 4). This suggests that the *cis*-located tailoring Dip20 methyltransferase and Dip5 amidotransferase may not be random insertions but functionally significant tailoring enzymes, as their presence is conserved across different species between *A. azurea* and *S. caatingaensis*.^48^

**Figure 4:**
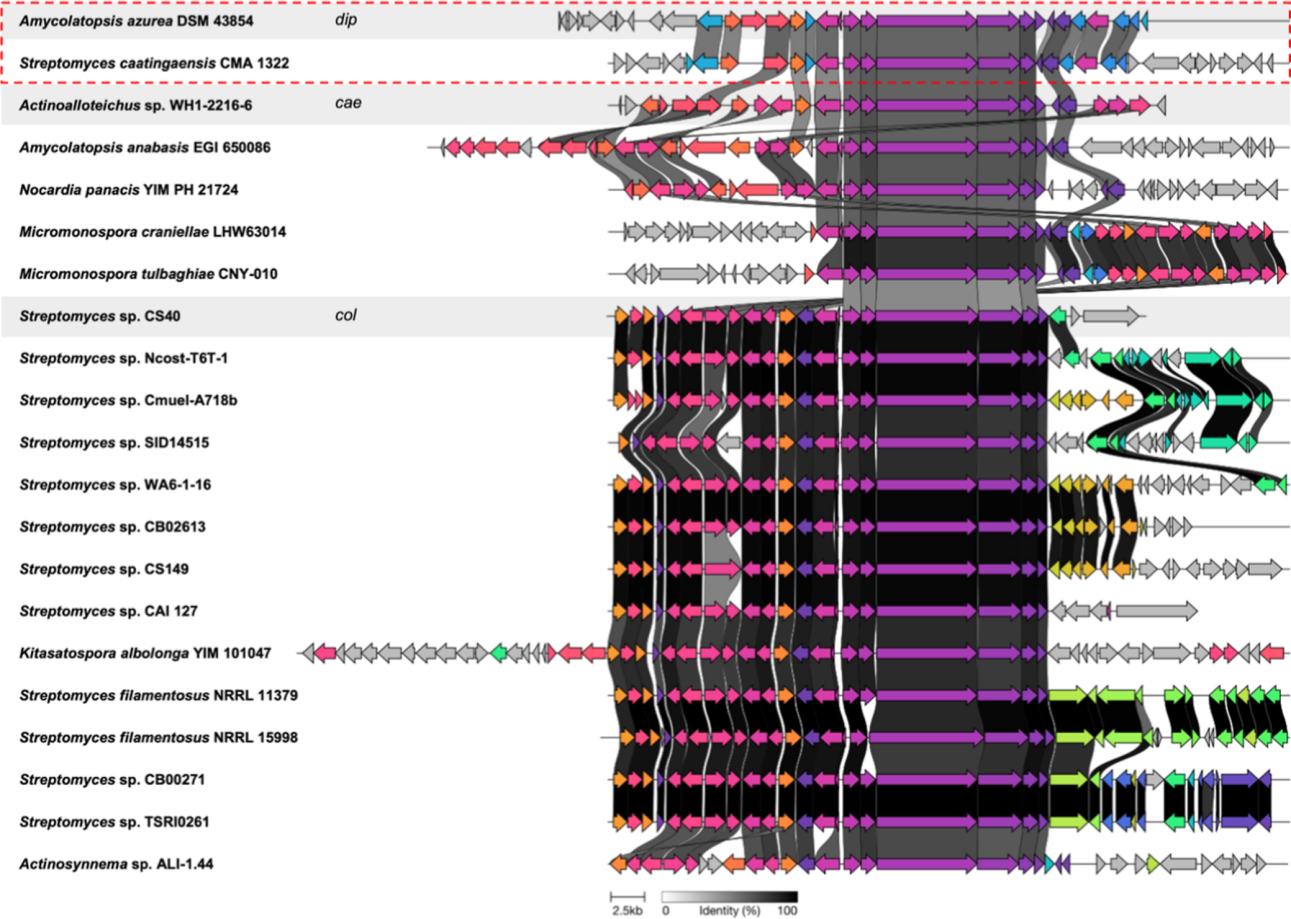
Clinker alignment, accessed through the CAGECAT Web server, of BGCs that are predicted to encode novel 2,2′-BP natural products from unreported bacterial strains.^49^ The clusters highlighted in the gray boxes represent the reference *dip*, *cae*, and *col* BGCs. The genes within the red dashed box correspond to the putative dip BGC found in *Amycolatopsis azurea* DSM 43854 (this paper) and a potential new producer, *Streptomyces caatingaensis* CMAA 1322. Genes that are homologous are color-coded similarly, and greyscale connections between genes further indicate >50% sequence identity.

Our genome mining analysis also highlights the variation in tailoring modifications around the 2,2′-BP core, suggesting further potential for more structural diversity among 2,2′-BP analogs (Figure 4). As demonstrated by the dipyrimicins and other members of the 2,2′-BP family, these tailoring modifications play a crucial role in modulating biological function. This underscores a key limitation in traditional genome mining, which relies primarily on the identification of conserved biosynthetic genes. This is corroborated by our ML prediction of just the 2,2′-BP forming genes with an average antibacterial activity prediction of 50%, indicating that the presence of the 2,2′-BP core may not be solely predictive of the antimicrobial potential. Therefore, genome-mining approaches could benefit from using our ML method in tandem by considering the total set of genes within the BGC, allowing for more informed prioritization for natural product discovery.

Apart from 2,2′-BP biosynthesis, our ML approach can be integrated with targeted genome mining to prioritize other natural product scaffolds and bioactive features,^50^ including activity-associated genes.^4^ However, a key challenge in enhancing ML-based predictions is the limited availability of high-quality data. We expect that as we are able to grow our training set and eventually incorporate quantitative rather than qualitative activity labels, the accuracy and utility of our machine learning model will improve, further accelerating the discovery of novel bioactive natural products. Therefore, it is beneficial for the broader natural product community for genome assemblies, BGC annotations, and the activities, including lack of activity, of the linked natural products to be reported in publications and shared public databases such as MIBiG and NPAtlas for improving our ML method.^29,51^

## Conclusion

We used ML-guided genome mining in combination with bioactivity-guided fractionation to guide the isolation of two antibacterial compounds, dipyrimicins A (1) and B (2). While dipyrimicins were originally isolated from a related *Amycolatopsis* sp. K16–0194, this marks the first time that 2,2′-BP family compounds and the corresponding BGC have been identified from *A. azurea* DSM 43854. Among the BGCs within *A. azurea* DSM 43854 predicted to encode an antimicrobial natural product, we deorphaned a cluster with 76% activity probability as the *dip* BGC. Importantly, this represents the first example of a BGC identified using this ML method that was not part of the model’s training set. The *dip* BGC shares only 40–52% similarity to previously characterized 2,2′-BP BGCs, validating the model’s ability to predict bioactive compounds from previously uncharacterized BGCs.

To confirm the link between dipyrimicins and the *dip* BGC, we performed in silico sequence and structural analyses, identifying key tailoring enzymes, including an O-methyltransferase and amidotransferase whose functions align with the structural modifications observed in dipyrimicins A and B. Specifically, the dipyrimicins differ in the presence of a carboxylic or amido group, and our findings suggest that C7′-amidation, catalyzed by Dip5, weakens antibacterial activity. This highlights the importance of tailoring enzymes modulating bioactivity. However, the biological advantage of C7′-amidation remains unknown, and further studies are needed to determine its potential significance in the physiology and ecology of *A. azurea* DSM 43854. Also, we expanded our ML-guided genome mining to additional 2,2′-BP BGCs and identified novel tailoring enzymes that could further contribute to structural and functional diversity within this class of natural products. Future studies can benefit from incorporating both specific tailoring genetic information and SAR trends to refine ML methods and provide insight for synthetic biology efforts to produce a more potent antibiotic natural product.

This study demonstrates how ML-guided approaches can be leveraged for natural product discovery. While the effort described in this paper resulted in the discovery of a known compound, it enabled the deorphanization of its cognate BGC. Another limitation of this study is that the putative dipyrimicin BGC is similar to two BGCs in the model’s training set: the caerulomycin and collismyicn BGCs. A more stringent test of this method would be to see if it can guide isolation of an active compound whose BGC has little or no similarity to any training set of BGCs. However, we believe that this approach has broad applicability in prioritizing strains capable of producing bioactive natural products, particularly in less-studied strains, where genomic information is available but their metabolites remain uncharacterized. We will continue to apply our ML genome mining pipeline, with the goal of identifying novel bioactive secondary metabolites. Additionally, future studies will focus on integrating this approach with other natural product discovery strategies, such as high-throughput activity screenings and heterologous expression, for more targeted prioritization.

## Experimental Section

### General Experimental Procedures and Materials

*A. azurea* DSM 43854 strain used throughout this study was obtained from the German Collection of Microorganisms and Cell Cultures (DSMZ). The *B. spizizenii* ATCC 6633 indicator strain was obtained from Fisher Scientific. The remaining indicator strains used for bioactivity studies, including *E. coli* MG1655 ATCC 700926, *P. aeruginosa* ATCC 27853, and *S. aureus* ATCC 25923, were obtained from the American Type Culture Collection (ATCC). BD Difco ISP2 and ISP4 media for bacterial growth were purchased from Fisher Scientific along with HyperSep C18 cartridges. Oxoid TSB media used for the MIC assay and the HyperSIL GOLD C18 columns were acquired from Thermo Scientific. All solvents used for organic extractions, chromatography, and spectrometry experiments were HPLC and LC–MS grade materials and were purchased from Fisher Scientific and Sigma-Aldrich. NMR solvents were purchased from Sigma-Aldrich.

### Genome Mining and BGC Activity Prediction

The genome sequence of *A. azurea* DSM 43854 (NCBI entry GCF_001995215.1) was downloaded from the NCBI database. As the machine learning algorithm by Walker and Clardy was trained on gene annotations by antiSMASH 5 and resistance gene identifier (RGI) version 5, these same versions were used for the activity prediction.^4,8,9^ For the BGCs of interest, no notable differences were observed between the results from antiSMASH versions 5 and 7. Therefore, AntiSMASH 5 was retained for a majority of the bioinformatic analysis to ensure consistency.

The BGCs of collismycin A (Genbank accession no. HE575208.1)^21^ and caerulomycin A BGC (Genbank accession no. JF419316.2)^26^ were aligned with the putative dipyrimicin BGC using clinker through the CAGECAT Web server (https://cagecat.bioinformatics.nl/).^49^ To identify other putative producers of 2,2′-bipyridine-containing compounds, using the 2,2′-BP forming genes as a query, a combination of cblaster,^52^ Enzyme Function Initiative-Enzyme Similarity Tool (EFI-EST),^52,53^ and AntiSMASH 7 (BLAST against antiSMASH-database)^54^ were used. Clinker, through the CAGECAT Web server, was used to align and confirm that each BGC, at the least, contains the conserved 2,2′-BP genes.^49^

For the structural analysis, the AlphaFold Server and its default parameters were used to generate the AlphaFold3 3D structures from the amino acid sequences of the *dip* genes.^36,55^ Structural models relevant to our analyses were identified and structurally aligned using the FoldSeek (TM-align mode) and FoldMason Web server (https://search.foldseek.com).^37,56^ All structural models and figures were generated and visualized with PyMOL molecular graphics software (version 2.0, Schrödinger).

### Fermentation

A frozen glycerol stock of *A. azurea* DSM 43854 was streaked onto ISP2 agar plates (4.0 g of yeast extract, 10.0 g of malt extract, 4.0 g of glucose, and 20.0 g of agar per 1L of H_2_O) at 30 °C until individual colonies were visible. A single colony was used to inoculate 5 mL of liquid ISP2 medium as seed culture and incubated at 30 °C overnight (200 rpm). The seed culture was used to inoculate ISP4 agar plates (10.0 g soluble starch, 1.0 g K_2_HPO_4_, 1.0 MgSO_4_•7H_2_O, 0.1 mg MnCl_2_•4H_2_O, 0.1 mg ZnSO_4_•7H_2_O, and 20.0 g agar per 1L H_2_O) and incubated at 30 °C for 14 days.

### Extraction and Isolation

After 14 day fermentations, the agar plates were frozen (−20 °C) overnight before thawing for >5 h prior to extraction. The agar plates were then soaked in equal volumes of ethyl acetate (1L per 20 plates) and deionized water and sonicated for ∼ 1 h. The liquid extract was separated from the solid agar via vacuum filtration, and the ethyl acetate layer was further separated via liquid–liquid extraction. The organic fraction was concentrated in vacuo to yield around 94 mg of crude extract per 1L of media. The crude sample was redissolved in 25% acetonitrile (ACN) for further analysis and isolation.

### Bioactivity-Guided Isolation

The crude extract underwent further separation through rounds of solid-phase extraction (SPE), flash chromatography, and semipreparative HPLC. Dipyrimicins A and B were identified as bioactive compounds of interest via bioactivity-guided fractionation in which each chromatography fractions obtained were concentrated in vacuo and then subjected to a bioassay initially against *B. spizizenii* ATCC 6633. Those showing activity (any observed zone of inhibition greater than the 25% ACN negative control) were further fractionated to obtain individual bioactive metabolites. SPEs were performed using HyperSep C18 Cartridges (5000 mg of bed weight). The cartridge was washed with 2–3 column volumes (CV) of 100% Methanol and equilibrated with 2 CVs of water before loading the crude sample. Analytes were then eluted from the column using 25%, 50%, 75%, and 100% ACN. Fractions collected at 50% ACN displayed a large zone of inhibition and were subjected to flash chromatography. Büchi Pure C-850 FlashPrep system equipped with UV and ELSD detectors and FlashPure Ecoflex C18 cartridge (50 μm, spherical, 4 g) were used in linear gradient from 0 to 100% ACN and decreasing volumes of H_2_O in 10 min. Bioactivity was observed on fractions eluting at around 3 min, which were advanced for further HPLC purifications. These were carried out on a Thermo Fisher Vanquish HPLC system equipped with a photodiode array detector and an automated fraction collector using a Hypersil GOLD C18 column (3 μm, 4.6 × 100 mm) at a flow rate of 1 mL/min using a linear gradient from 0 to 100% solvent A over 10 min (A = 95:5 ACN/H_2_O + 0.1% (v/v) formic acid (FA); B = 95:5 H_2_O/ACN +0.1% (v/v) FA). Individual peaks corresponding to active compounds 1 (1.1 mg yield per L) and 2 (0.5 mg yield per L) were collected at around 5.5 to 6.5 min. These reported isolated yields are for one batch of isolation. Large-scale growths were carried out in ten batches of 1L ISP4 media, and extraction and isolation were conducted as previously described.

### Characterization

UV–vis spectra were recorded from 190–500 nm on a Thermo Fisher Vanquish HPLC system equipped with a photodiode array detector. An analytical Hypersil GOLD C18 column (3 μm, 4.6 mm × 100 mm) was used to resolve samples at a flow rate of 1 mL/min with a linear gradient from 0 to 100% solvent A (A = 95:5 ACN/H_2_O + 0.1% (v/v) FA; B = 95:5 H_2_O/ACN +0.1% (v/v) FA) in 10 min. ESI-HRMS were measured on a LTQ-Orbitrap 3 XL at the Vanderbilt University Mass Spectrometry Core Lab. Data was acquired in positive ion mode between 100 and 2000 *m*/*z*. NMR spectra, including ^1^H, ^1^H–^1^H COSY, ^1^H–^13^C HSQC, and ^1^H–^13^C HMBC experiments, were recorded in CD_3_OD on a 600 MHz NMR spectrometer (Bruker) at the Vanderbilt University Small Molecule NMR Facility Core.

### Antimicrobial Assays

The minimum inhibitory concentration (MIC) was defined as the lowest concentration of dipyrimicin that inhibited visible growth after incubation. MICs to the indicator strains *B. spizizenii* ATCC 6633, *E. coli* MG1655 ATCC 700926, *P. aeruginosa* ATCC 27853, and *S. aureus* ATCC 25923 were determined using the broth microdilution method. The indicator strains were grown in Tryptic Soy Broth (TSB) overnight at 30 °C with shaking. Dipyrimicins A and B were prepared as 2-fold serial dilutions from 256 to 0.5 μg/mL in a 96-well plate using TSB with a total volume of 50 μL per well. The inoculum was prepared according to Kadeřábková et al.^57^ An equal volume of inoculum (50 μL per well) was added to reach the final test concentrations of 128 μg/mL to 0.25 μg/mL. The plates were incubated at 30 °C for 24 h, and the absorbance at OD_600_ was monitored using the Varioskan LUX Multimode Microplate Reader. Tetracycline was used as a positive control and yielded MIC values similar to previously reported values.^58–60^ Each sample concentration was measured in triplicate.

## Supplementary Material



## Notes

HPLC chromatogram, HR-ESI-MS spectrum, NMR of dipyrimicins A and B; structural alignment of putative *dip* biosynthetic enzymes with top hits from FoldMason and FoldSeek; BGCs in *A. azurea* DSM 43854 as analyzed from antiSMASH 5; machine-learning-predicted probability scores of the *A. azurea* BGCs; top FoldSeek structural search hits for the putative hydroxylase, amidotransferase, synthetase/ligase, and hydrolase involved in dipyrimicin biosynthesis; antibacterial activities of dipyrimicins A and B reported as MIC; and machine-learning antibacterial activity probability of BGCs encoding 2,2′-bipyridine genes (PDF)
