# Supplementary material for: AI-Assisted Isolation of Bioactive Dipyrimicins from Amycolatopsis azurea and Identification of Their Corresponding Dip Biosynthetic Gene Cluster
Source: J Nat Prod. 2026 Mar 26;89(4):1238–48. doi: 10.1021/acs.jnatprod.6c00057 (PMC13122635; doi:10.1021/acs.jnatprod.6c00057)
Supplement: Supplementary file 1 [file np6c00057_si_001.pdf]

Supporting Information for

AI-assisted isolation of bioactive Dipyrimicins from *Amycolatopsis azurea* and identification of its corresponding *dip* biosynthetic gene cluster

Christine Mae F. Ancajas,[1†] Isra Shuster,[1,2†] Allison S. Walker[1,2,3*]

[1] Department of Chemistry, Vanderbilt University, 1234 Stevenson Center Lane, Nashville, TN 37240, United States
[2] Department of Biological Sciences, Vanderbilt University, VU Station B, Box 35-1634, Nashville, TN 37235, United States
[3] Department of Pathology, Microbiology, and Immunology, 1211 Medical Center Drive, Vanderbilt University Medical Center, Nashville, TN 37232, United States

[†] authors contributed equally
[*] address correspondence to: Allison Walker (allison.s.walker@vanderbilt.edu)

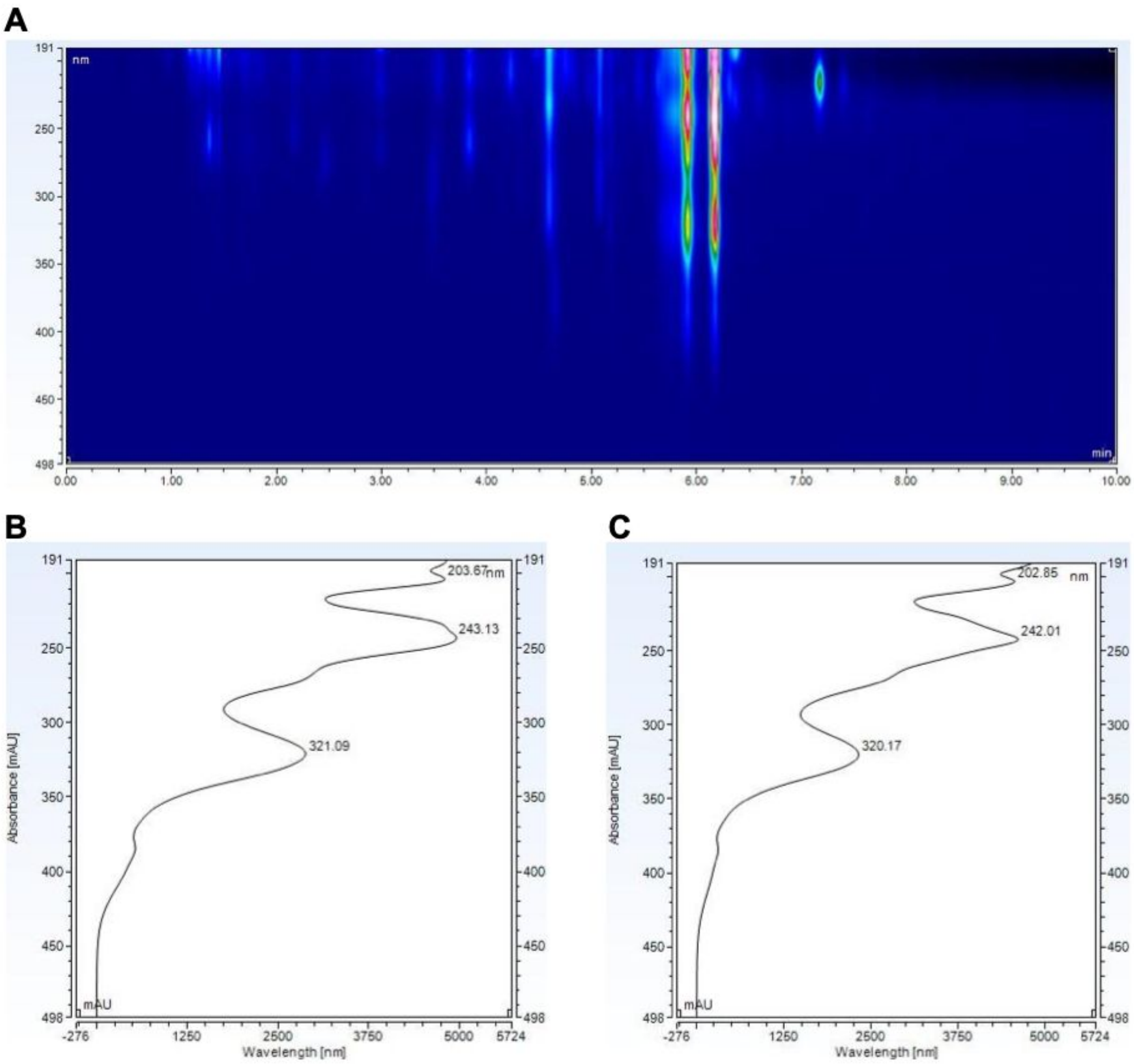

Figure S1. (A) HPLC chromatogram and UV-Vis spectrum of *A. azurea* DSM 43854 culture extract showing typical UV-Vis spectrum for 2,2′-bipyridines (**B**) Dipyrimicin A (compound **1,** λ = 204nm, 243nm, 268nm, and 321nm, retention time (RT) at ~5.8 min) and (**C**) Dipyrimicin B (compound **2,** λ = 203nm, 242nm, 268nm, and 320nm, RT at ~6.2 min).

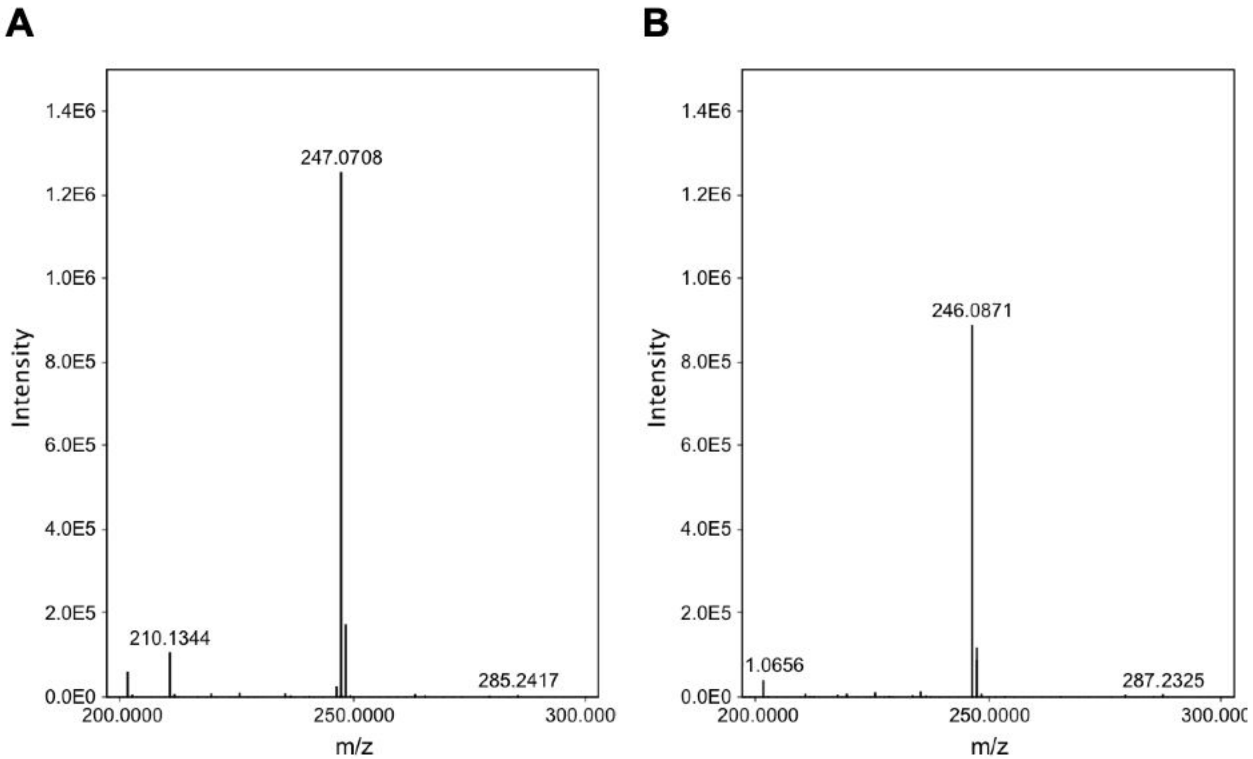

Figure S2. HR-ESI-MS spectrum of **(A)** Dipyrimicin A (compound **1,** m/z $[M+H]^+$ obsvd. 247.0708, calcd. for $C_{12}H_{10}N_2O_4$, 247.0719) and (**B**) Dipyrimicin B (compound **2,** m/z $[M+H]^+$ obsvd. 246.0871, calcd. for $C_{12}H_{11}N_3O_3$, 246.0879)

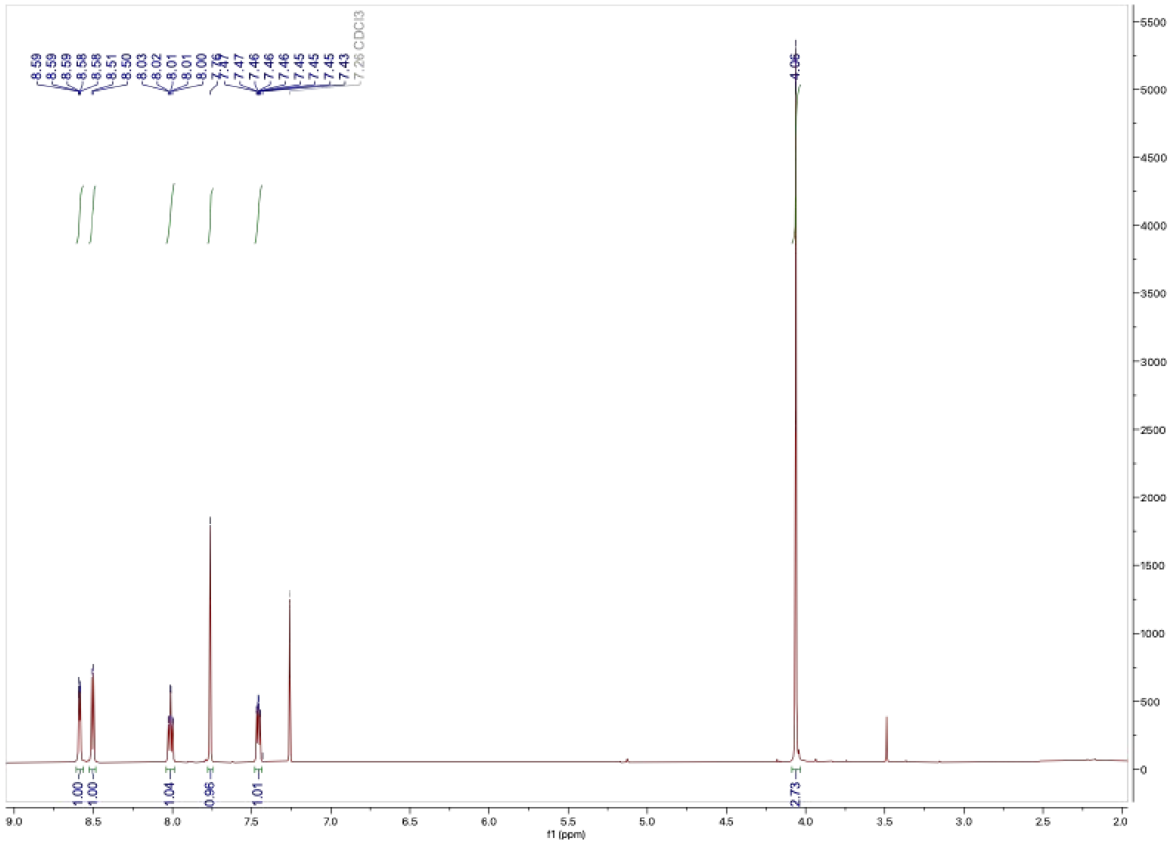

Figure S3. ^{1}H NMR (600 MHz) spectrum of dipyrimicin A (**1**) in $CDCl_3$

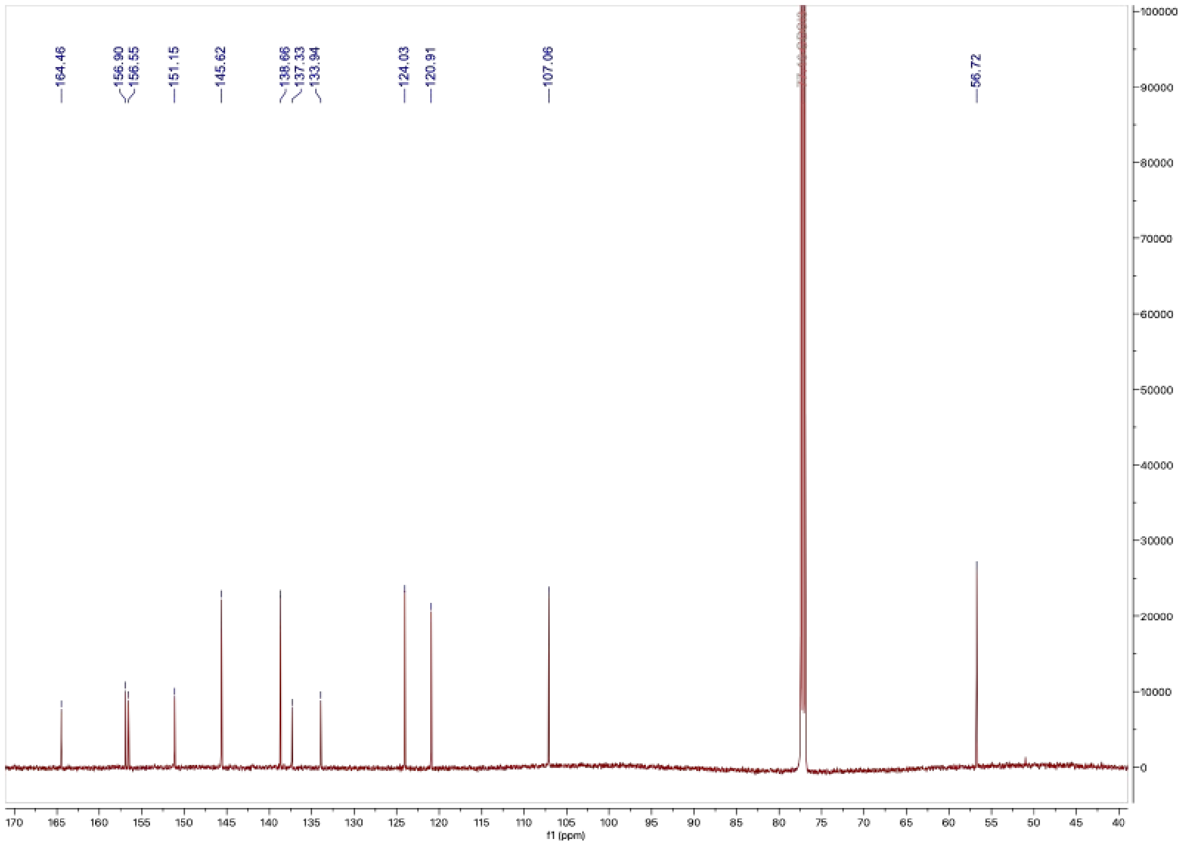

Figure S4. ^{13}C NMR (150 MHz) spectrum of dipyrimicin A (**1**) in $CDCl_3$.

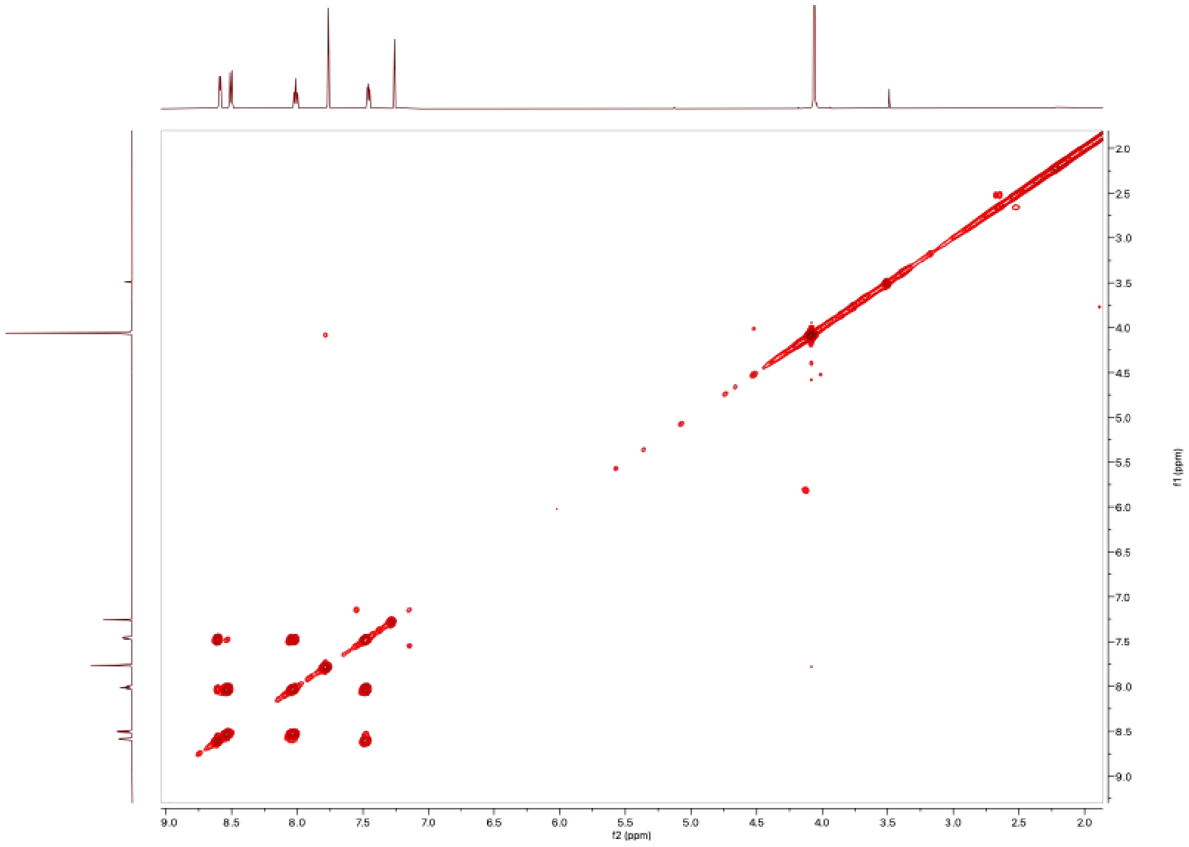

Figure S5. ^{1}H-^{1}H COSY NMR spectrum of dipyrimicin A (**1**) in $CDCl_3$.

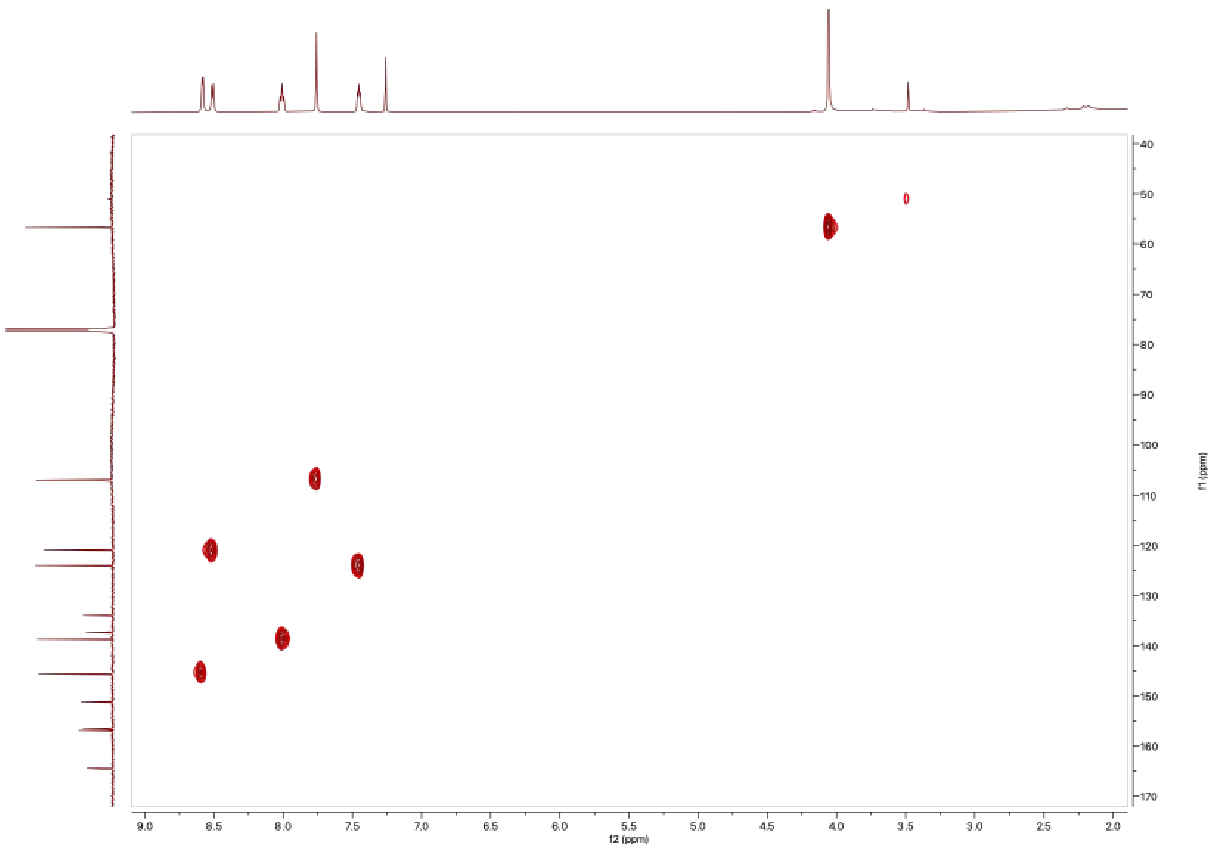

Figure S6. ^{1}H-^{13}C HSQC NMR spectrum of dipyrimicin A (**1**) in $CDCl_3$.

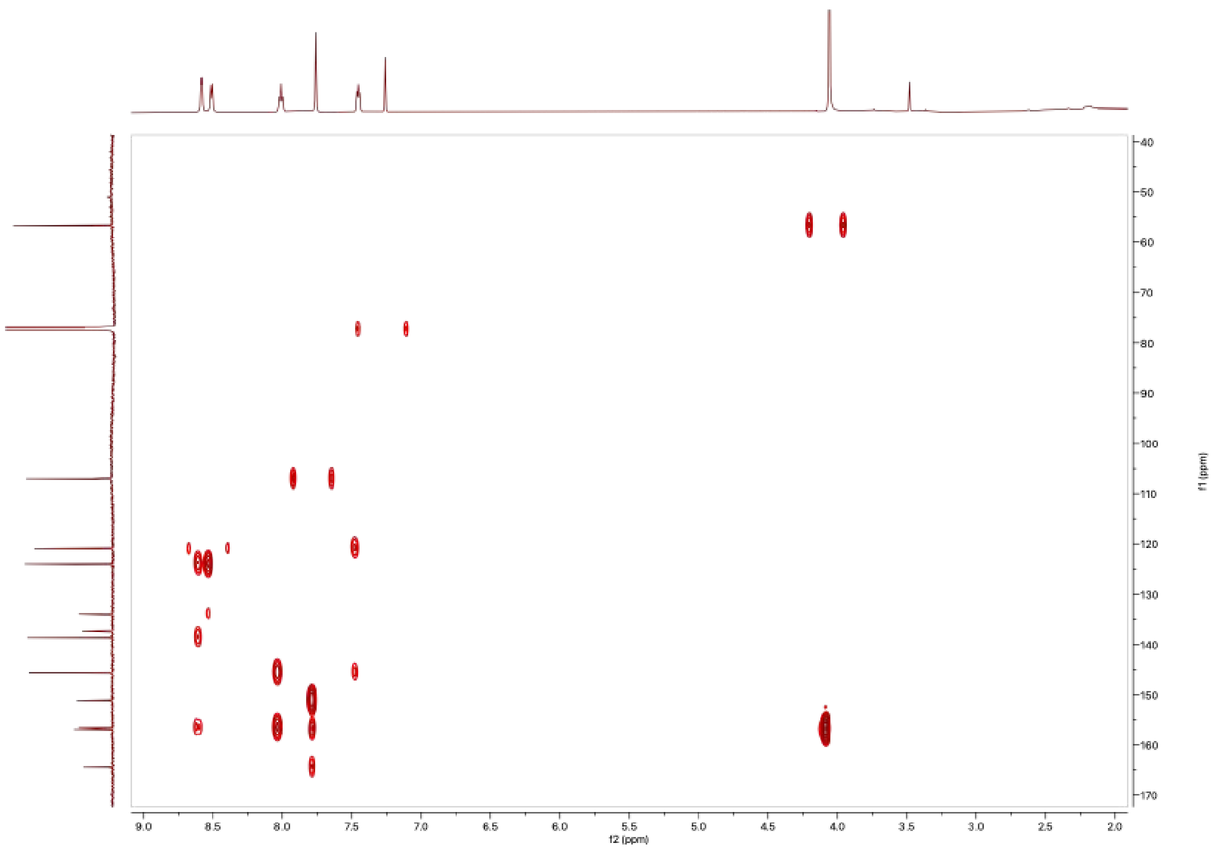

Figure S7. ^{1}H-^{13}C HMBC NMR spectrum of dipyrimicin A (**1**) in $CDCl_3$.

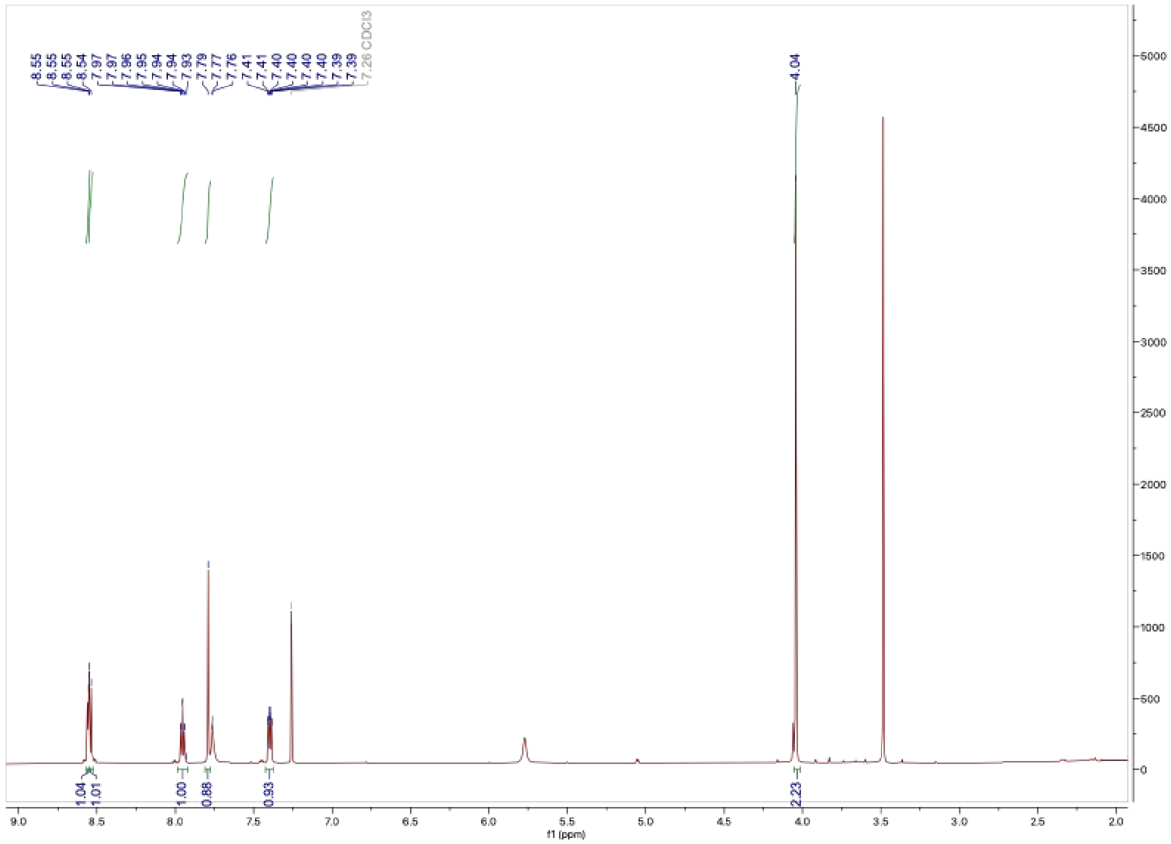

Figure S8. ^{1}H NMR (600 MHz) spectrum of dipyrimicin B (**2**) in $CDCl_3$.

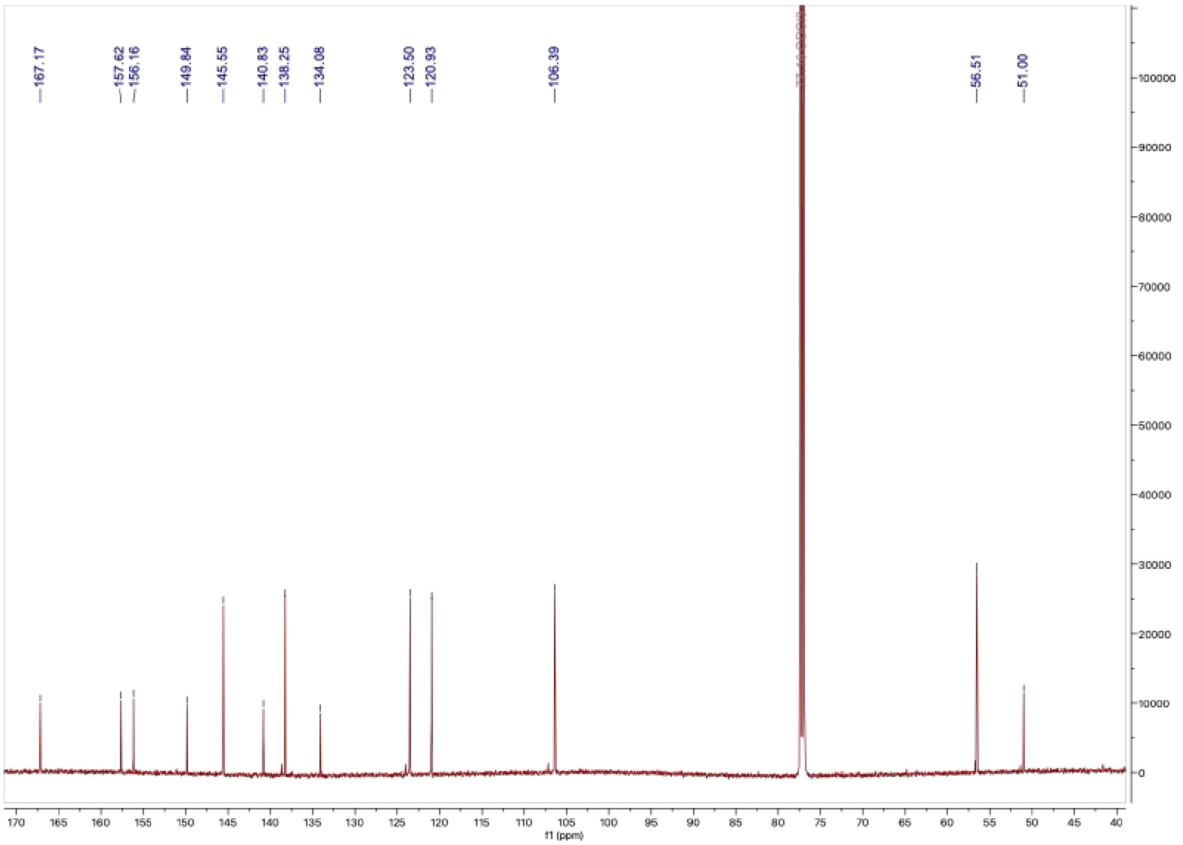

Figure S9. ^{13}C NMR (150 MHz) spectrum of dipyrimicin B (**2**) in $CDCl_3$.

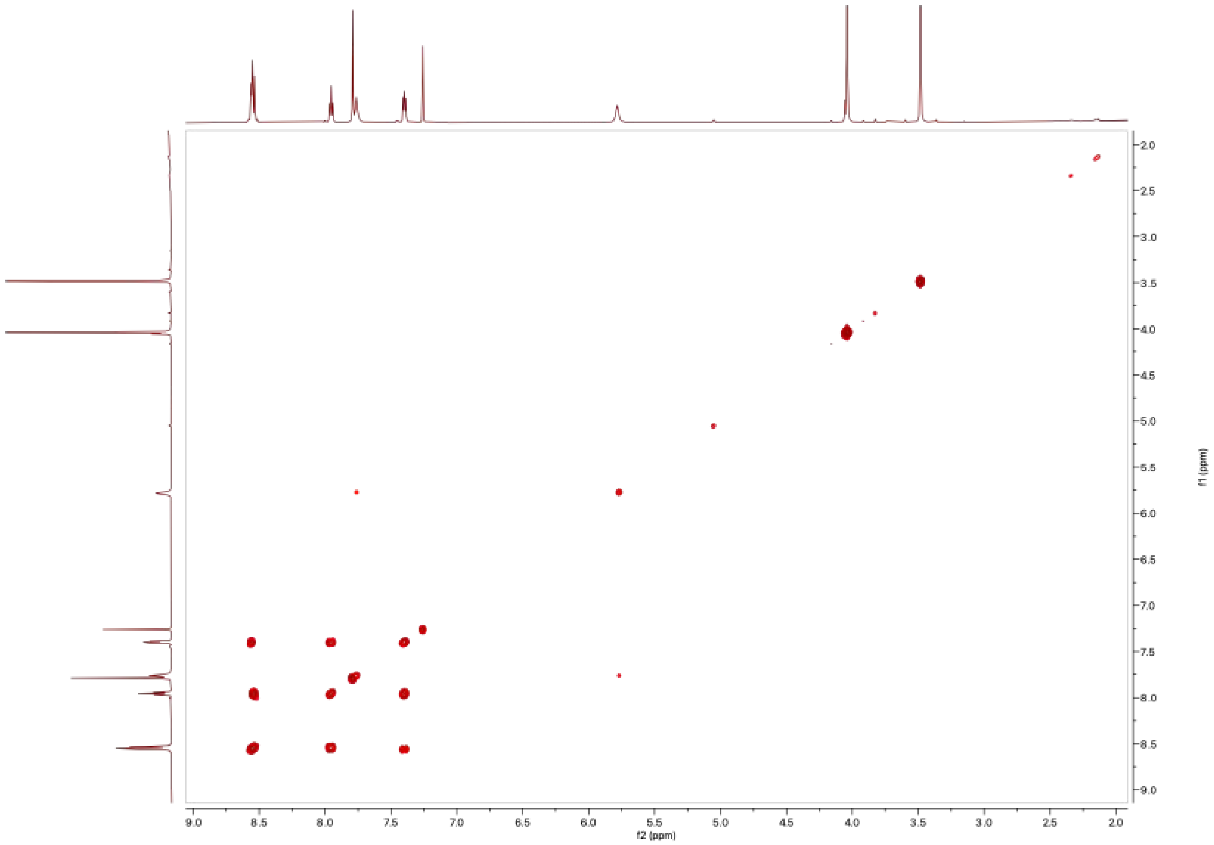

Figure S10. ^1H-^1H COSY NMR spectrum of dipyrimicin B (**2**) in $CDCl_3$.

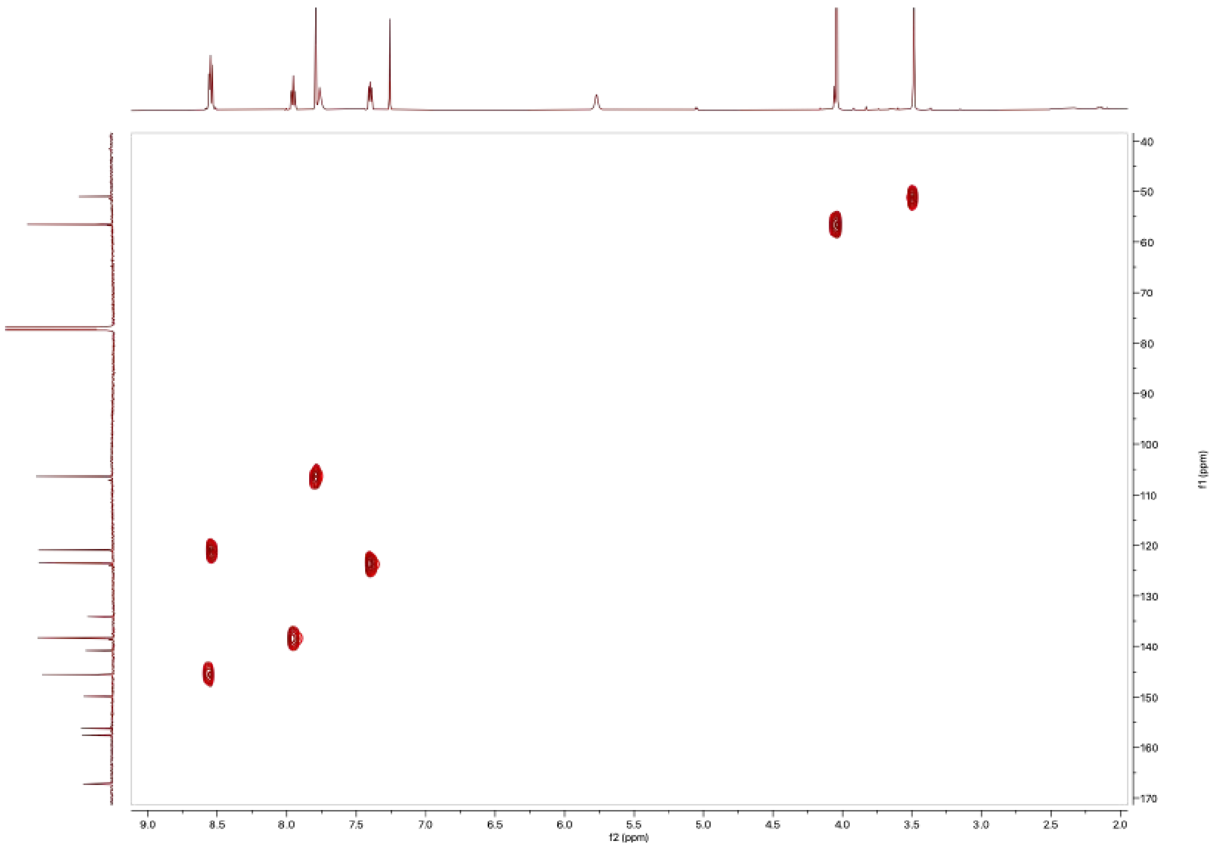

Figure S11. ^{1}H-^{13}C HSQC NMR spectrum of dipyrimicin B (**2**) in $CDCl_3$.

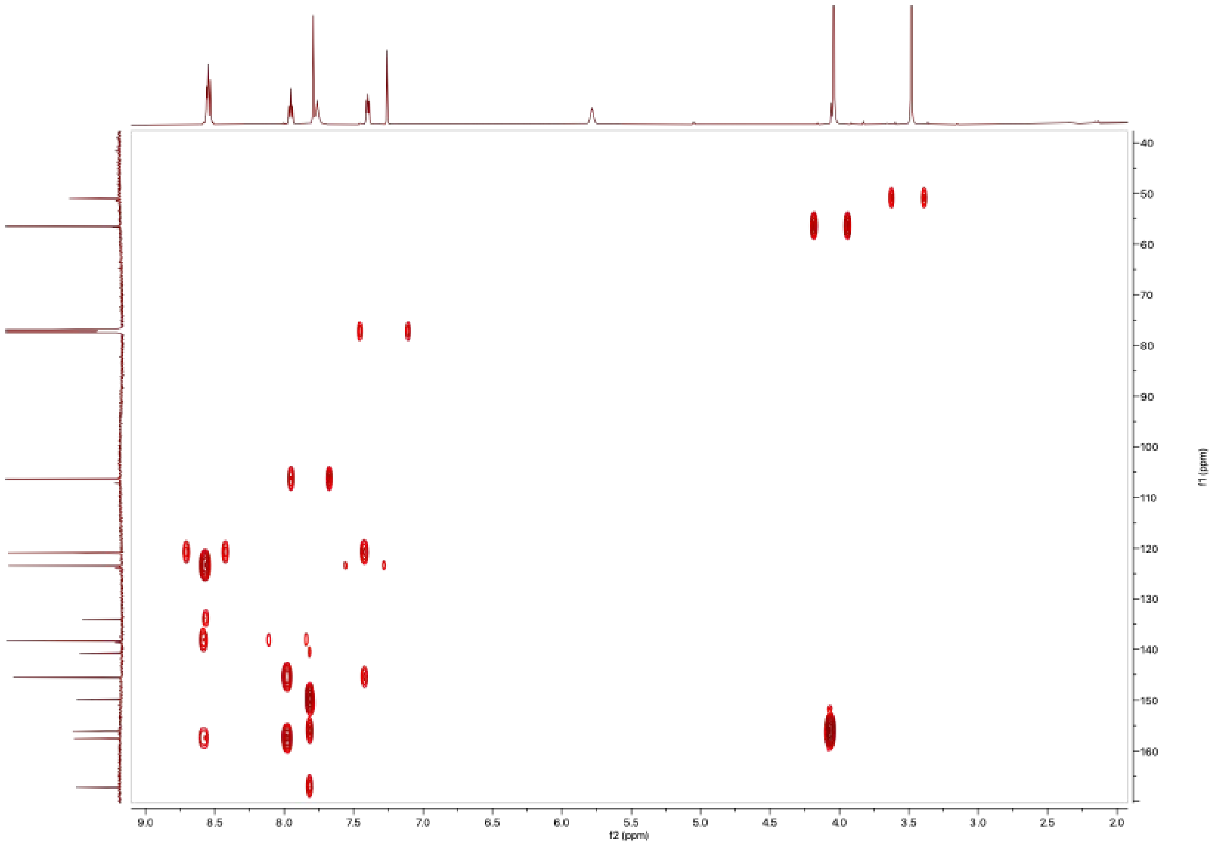

Figure S12. ^{1}H-^{13}C HMBC NMR spectrum of dipyrimicin B (**2**) in $CDCl_3$.

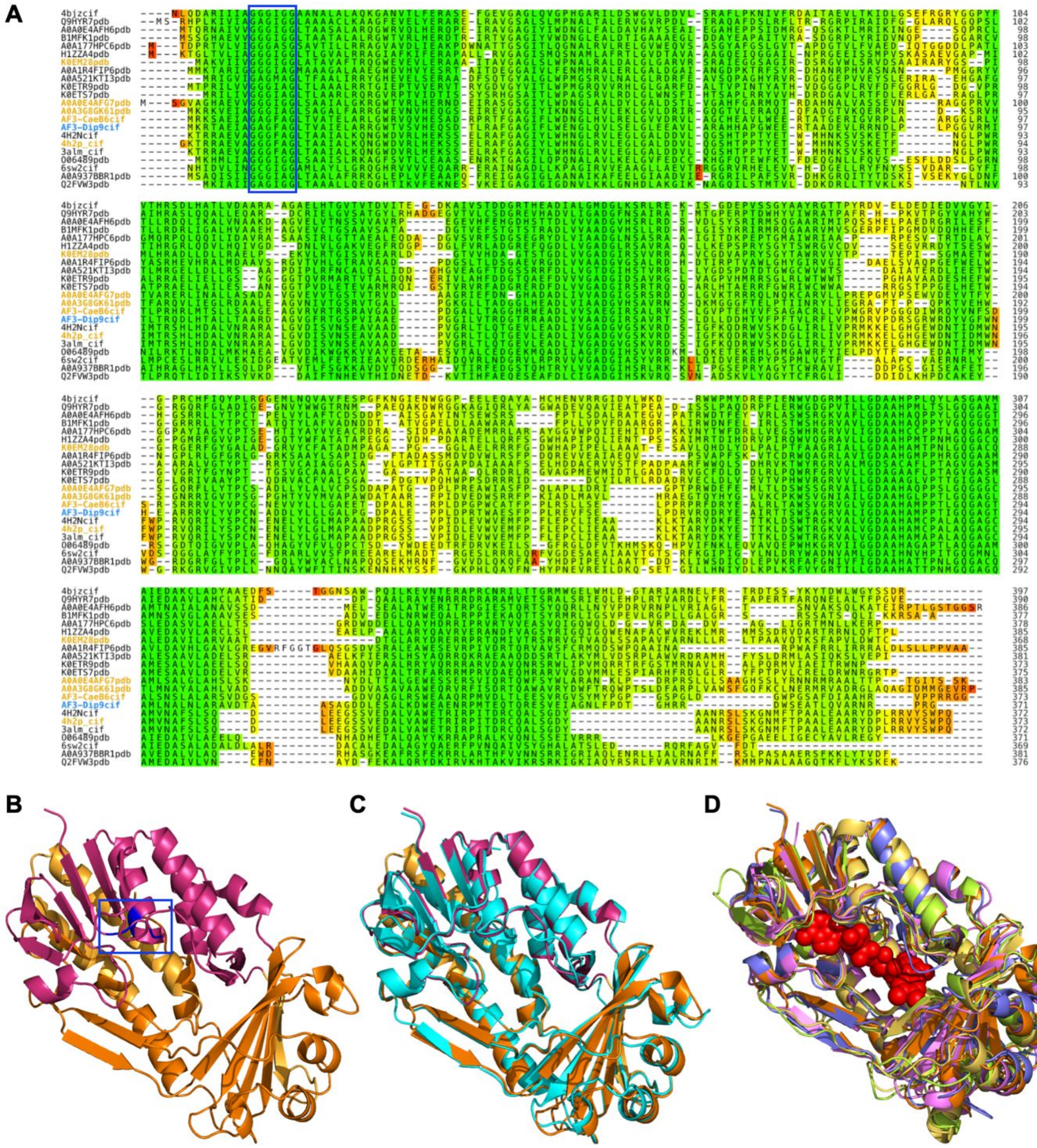

Figure S13. Multiple structural alignment (MSTA) of Dip9 and related hydroxylases. A) FoldMason MSTA of the Dip9 structure along with the top FoldSeek hits from different databases (**Table S4**). Residues are shaded by the LDDT score of their alignment column (color scheme: red 0%, green 100%). Highlighted in the blue box is the conserved "GxGxxG" motif found among flavin-containing oxygenases.[1] B) The AlphaFold-predicted structure of Dip9. Highlighted in pink and dark orange are the putative FAD-binding and substrate-binding domains, respectively. The glycine-rich motif is depicted in blue within the box. C) Superimposition of the predicted structures of Dip9 and CaeB6 (cyan) with RMSD of 0.70 Å. D) Superimposition of Dip9 and the top five FoldSeek hits from different databases with RMSD values ranging from 0.86 to 2.79 Å (**Table S4**). The representative FAD is depicted as red spheres (general location of FAD in PDBs 4H2P), highlighting that the related hydroxylases have similar sites for FAD- and substrate-binding.

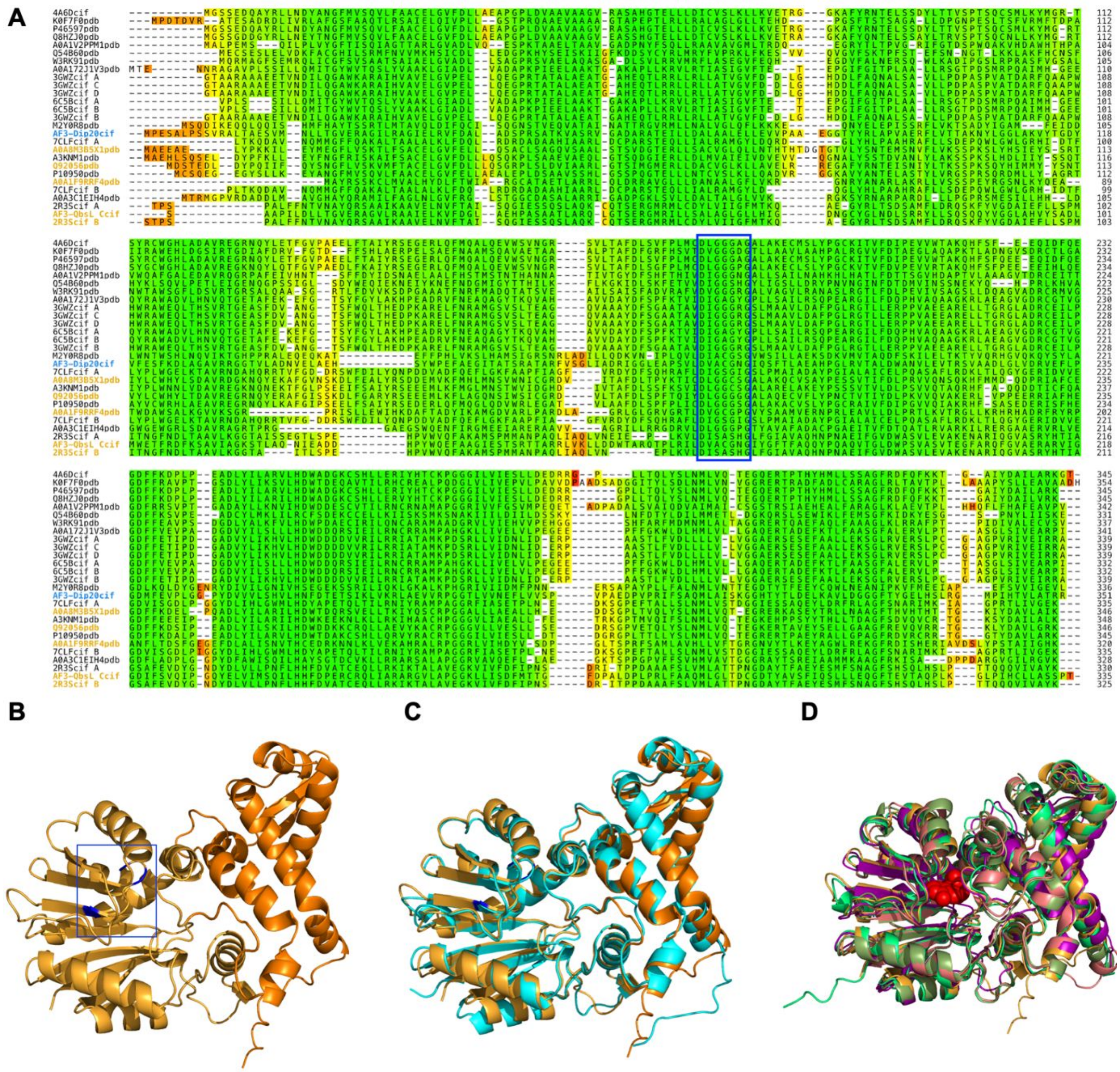

Figure S14. Multiple structural alignment (MSTA) of Dip20 and related methyltransferases. A) FoldMason MSTA of the Dip20 structure along with the top FoldSeek hits from different databases (**Table S5**). Residues are shaded by the LDDT score of their alignment column (color scheme: red 0%, green 100%). Highlighted in the blue box is the “DxGxGxG” or “GxG” fingerprint, a SAM-binding motif found among class I methyltransferases. B) The AlphaFold-predicted structure of Dip20. The C-terminal domain (light orange) contains the pocket for SAM- and substrate-binding while the N-terminal domain is for dimerization. The glycine-rich motif is depicted in blue within the box. C) Superimposition of the predicted structures of Dip20 and the C-terminal domain of QbsL (cyan) (RMSD of 1.72 Å). D) Superimposition of Dip20 and the top five FoldSeek hits from different databases with RMSD values ranging from 1.47 to 2.43 Å (**Table S5**). The representative SAM molecule is depicted as red spheres (from 2R3S PDB).

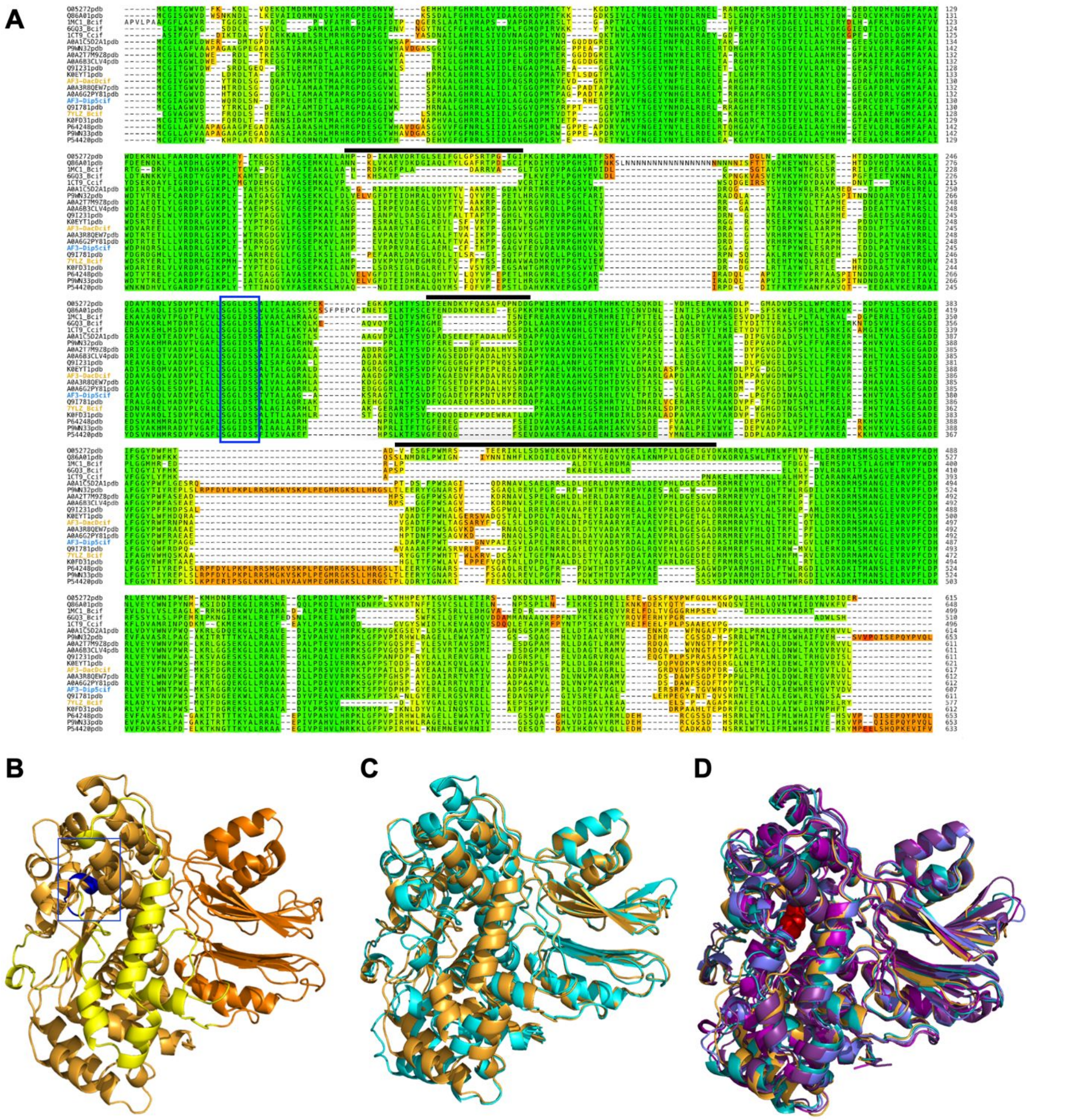

Figure S15. Multiple structural alignment (MSTA) of Dip5 and related amidotransferases. A) FoldMason MSTA of the Dip5 structure along with the top FoldSeek hits from different databases (**Table S6**). Residues are shaded by the LDDT score of their alignment column (color scheme: red 0%, green 100%). Highlighted in the blue box is the conserved "SGGLDS" motif among amidotransferases. Dip5 and several other related proteins reveal inserted regions (black bars) that are absent when compared to the glutamine-dependent asparagine synthetase, AsnB, from *Escherichia coli* (PDB 1CT9). B) The AlphaFold-predicted structure of Dip5. Highlighted in light and dark orange are the C-terminal synthetase and N-terminal glutaminase domains, respectively. Residues in yellow are the inserted regions not found in AsnB. The conserved motif involved in ATP-binding is depicted in blue within the box. C) Superimposition of the predicted structures of Dip5 and DacD (cyan) (RMSD of 0.53 Å). D) Superimposition of Dip5 and the top five FoldSeek

hits from different databases with RMSD values ranging from 0.57 to 1.01 Å (**Table S6**). The representative ATP molecule is depicted as red spheres (from 1CT9 PDB).

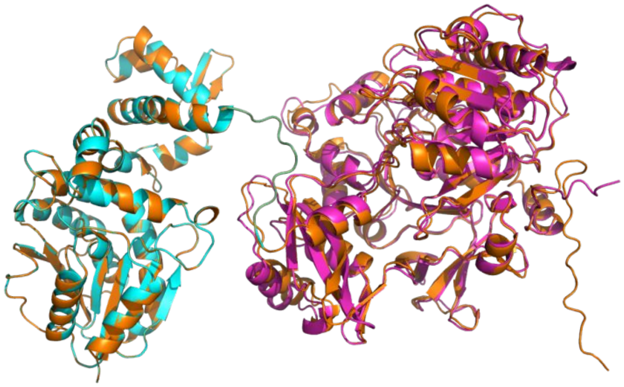

Figure S16. Superimposition of the AlphaFold-predicted structures of Dip21 (magenta) and Dip20 (cyan) to the N-terminal synthetase (RMSD of 0.76 Å) and C-terminal methylase (RMSD of 1.72 Å) domains of QbsL (orange), respectively.

Table S1. The biosynthetic gene clusters (BGCs) of secondary metabolites in *Amycolatopsis azurea* DSM 43854 analyzed from antiSMASH 5.

Cluster	Type	From	To	Most similar known BGC (% similarity)
1.1	CDPS	137,563	158,264	
1.2	terpene	210,895	233,051	geosmin (100%)
1.3	ectoine	256,222	266,611	ectoine (100%)
2.1	T1PKS	318	68,485	butyrolactol A (66%)
2.2	hglE-KS, T1PKS	108,031	158,424	rimosamide (14%)
2.3	lassopeptide	268,408	290,889	ansacarbamitocin A (12%)
2.4	NRPS, amglyccycl	324,333	378,452	acarbose (14%)
2.5	NRPS, T1PKS	378,800	487,216	caerulomycin A (52%)
2.6	NRPS	548,461	609,364	
2.7	T1PKS	610,946	657,329	kanamycin (1%)
5.1	lanthipeptide	90,029	112,572	Ery-9 / Ery-6 / Ery-8 / Ery-7 / Ery-5 / Ery-4 / Ery-3 (75%)
6.1	terpene	166,574	187,503	isorenieratene (42%)
7.1	NRPS-like	10,140	54,042	ansacarbamitocin A (4%)
10.1	NRPS	16,609	84,811	mirubactin (78%)
11.1	terpene	23,959	46,151	enduracidin (10%)
11.2	betalactone	66,497	91,707	
12.1	arylpolyene	78,681	119,832	kinamycin (5%)
15.1	NRPS	116,412	178,671	albachelin (100%)
16.1	bacteriocin	162,646	173,458	
16.2	terpene	364,878	386,005	vazabitide A (4%)
16.3	T1PKS	432,515	469,723	funisamine (22%)
20.1	other, arylpolyene	21,861	73,484	kedarcidin (19%)

22.1	NRPS-like	181,449	225,240	meilingmycin (3%)
23.1	NRPS, CDPS, T1PKS, T3PKS, terpene	1,589	122,641	diazepinomicin (17%)
24.1	terpene	65,035	86,174	2-methylisoborneol (100%)
24.2	NRPS-like, lanthipeptide	108,423	151,788	
24.3	NRPS	214,282	273,246	capreomycin IA / capreomycin IB / capreomycin IIA / capreomycin IIB (9%)
25.1	T3PKS, NRPS-like, NRPS	124,067	226,706	keratinimicin A / keratinimicin B / keratinimicin C / keratinimicin D (92%)
25.2	T1PKS, NRPS-like	233,345	279,671	polyoxypeptin (10%)
27.1	lanthipeptide	16,974	39,844	
28.1	T1PKS	3,842	49,676	amycolamycin A / amycolamycin B (31%)
28.2	T2PKS	134,047	206,532	actinorhodin (59%)
29.1	hglE-KS, T1PKS	14,179	64,527	rifamorpholine A / rifamorpholine B / rifamorpholine C / rifamorpholine D / rifamorpholine E (9%)
33.1	T1PKS, NRPS	1	46,481	chlortetracycline (5%)
45.1	T1PKS	1	26,493	

Table S2. Machine-learning predicted probability scores for various bioactivities of the AntiSMASH 5.0-annotated BGCs from *A. azurea DSM 43854*. The scores were obtained using three classifiers [extra trees, logistic regression, and support vector machine (SVM)] and were averaged for each activity classification. For reference, the average predicted activities of antibacterial compounds in the original held out dataset for this method are 0.59 (extra trees), 0.54 (logistic regression), 0.62 (SVM), not that these average include BGCs with no similarity to the training set, for which accuracy is low.

	Antibacterial				Anti-gram positive				Anti-gram negative				antifungal/antitumor/cytotoxic				Antifungal				Antitumor/cytotoxic			
Cluster	Tree	Log	SVM	Avg	Tree	Log	SVM	Avg	Tree	Log	SVM	Avg	Tree	Log	SVM	Avg	Tree	Log	SVM	Avg	Tree	Log	SVM	Avg
1.1	0.68	0.50	0.56	0.58	0.56	0.27	0.28	0.37	0.24	0.05	0.04	0.11	0.36	0.38	0.33	0.36	0.19	0.13	0.05	0.12	0.26	0.28	0.30	0.28
1.2	0.35	0.37	0.60	0.44	0.48	0.28	0.35	0.37	0.12	0.15	0.19	0.15	0.28	0.19	0.18	0.22	0.20	0.23	0.18	0.20	0.10	0.14	0.12	0.12
1.3	0.46	0.35	0.17	0.33	0.40	0.15	0.17	0.24	0.00	0.09	0.09	0.06	0.20	0.23	0.22	0.22	0.07	0.16	0.09	0.11	0.03	0.06	0.05	0.05
2.1	0.36	0.28	0.42	0.35	0.44	0.11	0.20	0.25	0.12	0.15	0.17	0.15	0.80	0.89	0.92	0.87	0.42	0.54	0.34	0.43	0.44	0.25	0.44	0.38
2.2	0.66	0.53	0.62	0.60	0.70	0.46	0.51	0.56	0.60	0.35	0.23	0.39	0.80	0.69	0.75	0.75	0.46	0.36	0.30	0.37	0.38	0.12	0.23	0.24
2.3	0.90	0.86	0.98	0.91	0.78	0.98	0.92	0.89	0.48	0.36	0.27	0.37	0.44	0.10	0.10	0.21	0.16	0.12	0.26	0.18	0.21	0.04	0.20	0.15
2.4	0.72	0.75	0.72	0.73	0.62	0.39	0.51	0.51	0.52	0.92	0.43	0.62	0.72	0.96	0.93	0.87	0.51	0.45	0.25	0.40	0.64	0.04	0.35	0.35
2.5	0.60	0.99	0.71	0.76	0.60	1.00	0.95	0.85	0.40	0.91	0.42	0.58	0.72	0.92	0.93	0.86	0.62	0.27	0.17	0.35	0.50	0.07	0.35	0.31
2.6	0.64	0.52	0.59	0.58	0.48	0.35	0.32	0.38	0.20	0.10	0.11	0.14	0.40	0.82	0.65	0.62	0.34	0.19	0.17	0.23	0.21	0.60	0.32	0.38
2.7	0.66	0.53	0.62	0.60	0.64	0.26	0.41	0.44	0.24	0.29	0.35	0.29	0.45	0.03	0.06	0.18	0.37	0.18	0.16	0.24	0.40	0.04	0.31	0.25
5	0.58	0.69	0.68	0.65	0.62	0.73	0.63	0.66	0.32	0.52	0.30	0.38	0.20	0.04	0.07	0.10	0.28	0.12	0.19	0.20	0.11	0.01	0.08	0.06
6	0.20	0.23	0.09	0.17	0.20	0.04	0.10	0.11	0.28	0.10	0.17	0.18	0.24	0.14	0.28	0.22	0.18	0.16	0.28	0.21	0.13	0.04	0.06	0.08
7	0.52	0.56	0.71	0.59	0.80	0.74	0.79	0.78	0.32	0.07	0.24	0.21	0.36	0.12	0.11	0.20	0.32	0.11	0.17	0.20	0.14	0.15	0.32	0.21
10	0.46	0.27	0.66	0.46	0.38	0.00	0.05	0.14	0.36	0.00	0.03	0.13	0.48	0.01	0.02	0.17	0.28	0.05	0.16	0.16	0.45	0.00	0.33	0.26
11.1	0.78	0.78	0.70	0.75	0.80	0.93	0.79	0.84	0.16	0.07	0.09	0.11	0.40	0.13	0.17	0.23	0.22	0.13	0.17	0.17	0.32	0.11	0.21	0.22
11.2	0.70	0.54	0.82	0.69	0.54	0.84	0.80	0.73	0.32	0.09	0.10	0.17	0.48	0.44	0.37	0.43	0.10	0.15	0.20	0.15	0.30	0.69	0.42	0.47
12	0.84	0.78	0.71	0.77	0.74	0.89	0.88	0.84	0.32	0.13	0.33	0.26	0.36	0.00	0.06	0.14	0.10	0.04	0.13	0.09	0.29	0.05	0.29	0.21
15	0.26	0.43	0.57	0.42	0.36	0.36	0.42	0.38	0.28	0.22	0.34	0.28	0.36	0.14	0.13	0.21	0.22	0.06	0.16	0.15	0.15	0.03	0.29	0.15
16.1	0.52	0.39	0.43	0.45	0.24	0.31	0.28	0.28	0.00	0.08	0.09	0.05	0.00	0.21	0.22	0.14	0.01	0.17	0.18	0.12	0.02	0.14	0.06	0.07
16.2	0.71	0.50	0.40	0.54	0.52	0.34	0.36	0.41	0.20	0.15	0.19	0.18	0.12	0.14	0.10	0.12	0.11	0.06	0.06	0.08	0.08	0.20	0.08	0.12
16.3	0.62	0.48	0.40	0.50	0.60	0.50	0.56	0.55	0.04	0.11	0.17	0.11	0.72	0.32	0.24	0.42	0.34	0.32	0.21	0.29	0.45	0.07	0.13	0.22
20	0.54	0.49	0.69	0.57	0.76	0.26	0.37	0.47	0.08	0.00	0.06	0.05	0.68	0.26	0.36	0.44	0.30	0.18	0.19	0.22	0.59	0.22	0.38	0.40
22	0.82	0.87	0.70	0.80	0.78	0.97	0.87	0.87	0.44	0.75	0.45	0.55	0.52	0.35	0.47	0.45	0.24	0.12	0.18	0.18	0.44	0.04	0.30	0.26
23	0.50	0.60	0.70	0.60	0.58	0.39	0.69	0.55	0.56	0.34	0.34	0.42	0.60	0.96	0.98	0.85	0.46	0.47	0.17	0.37	0.57	0.69	0.35	0.54
24.1	0.52	0.45	0.15	0.37	0.62	0.31	0.32	0.42	0.16	0.10	0.09	0.12	0.24	0.31	0.29	0.28	0.15	0.19	0.09	0.14	0.14	0.27	0.17	0.19
24.2	0.58	0.60	0.52	0.57	0.76	0.35	0.50	0.54	0.16	0.03	0.06	0.08	0.28	0.03	0.07	0.13	0.23	0.12	0.17	0.17	0.15	0.02	0.20	0.12
24.3	0.68	0.69	0.67	0.68	0.58	0.55	0.69	0.61	0.36	0.23	0.26	0.28	0.56	0.44	0.41	0.47	0.42	0.26	0.16	0.28	0.35	0.12	0.25	0.24
25.1	0.98	0.96	0.83	0.93	1.00	0.98	0.95	0.97	0.48	0.56	0.26	0.43	0.16	0.01	0.02	0.06	0.02	0.03	0.12	0.06	0.11	0.00	0.19	0.10
25.2	0.64	0.46	0.44	0.51	0.56	0.28	0.33	0.39	0.40	0.09	0.12	0.20	0.64	0.63	0.51	0.59	0.34	0.27	0.19	0.27	0.39	0.34	0.31	0.35
27	0.82	0.66	0.97	0.82	0.80	0.96	0.93	0.90	0.08	0.02	0.04	0.05	0.44	0.26	0.30	0.33	0.16	0.23	0.17	0.19	0.34	0.28	0.38	0.34
28.1	0.76	0.65	0.65	0.69	0.64	0.41	0.60	0.55	0.16	0.07	0.12	0.12	0.68	0.20	0.23	0.37	0.08	0.04	0.12	0.08	0.41	0.55	0.40	0.45
28.2	0.72	0.77	0.70	0.73	0.54	0.87	0.81	0.74	0.44	0.01	0.11	0.19	0.68	0.83	0.84	0.78	0.36	0.12	0.18	0.22	0.63	0.64	0.35	0.54
29	0.50	0.63	0.66	0.60	0.54	0.19	0.38	0.37	0.28	0.10	0.13	0.17	0.60	0.44	0.36	0.47	0.24	0.12	0.19	0.19	0.51	0.31	0.42	0.41
33	0.78	0.69	0.64	0.70	0.62	0.69	0.60	0.64	0.24	0.63	0.48	0.45	0.72	0.57	0.57	0.62	0.18	0.16	0.16	0.17	0.31	0.83	0.36	0.50
45	0.72	0.70	0.60	0.67	0.70	0.80	0.71	0.74	0.20	0.13	0.28	0.20	0.48	0.19	0.34	0.33	0.22	0.17	0.19	0.19	0.35	0.07	0.19	0.20

Table S3. List of genes in the dipyrimicin *dip* BGC, their proposed functions, and comparison to

the *cae* and *col* BGC.

dip gene	Size (AA)[a]	BLAST Hit protein [origin]	ID/SI[b] (%)	*cae* homolog	ID/SI[b] (%)	*col* homolog	ID/SI[b] (%)	proposed function
orf -3	123	YciI family protein [*Actinokineospora fastidiosa*]	90/95					
orf -2	425	RNA polymerase sigma factor [*Lentzea roselyniae*]	83/88					
orf -1	363	FAD-dependent oxidoreductase [*Amycolatopsis japonica*]	74/80					
1	201	TetR family transcriptional regulator [*Amycolatopsis alba*]	85/91	*caeI2*	33/50	*colI2*	30/58	regulator
2	135	hypothetical protein [*Amycolatopsis sp.* WAC 04169]	90/95					unknown
3	324	glycine betaine ABC transporter substrate-binding protein [*Amycolatopsis oliviviridis*]	96/97					putative transporter
4	822	ABC transporter permease subunit [*Amycolatopsis sp.* WAC 01416]	94/95					putative transporter
5	606	asparagine synthase (glutamine hydrolyzing) [*Fodinicola feengrottensis*]	62/73					putative amidotransferase
6	403	MFS transporter [*Streptomyces sp.* NPDC004629]	60/74	*caeH3*	43/60			putative transporter
7	611	ABC transporter ATP-binding protein [*Streptomyces sp.* NPDC004629]	68/80	*caeH1*	62/75	*colH1*	56/70	transporter
8	582	ABC transporter ATP-binding protein [*Streptomyces sp.* NPDC004629]	70/78	*caeH2*	63/75	*colH2*	59/71	transporter
9	369	NAD(P)/FAD-dependent oxidoreductase [*Amycolatopsis anabasis*]	65/71	*caeB6*	54/66			putative hydroxylase
10	231	alpha/beta fold hydrolase [*Streptomyces sp.* NPDC004629]	67/76					putative hydrolase
11b	542	(2,3-dihydroxybenzoyl)adenylate synthase [*Amycolatopsis anabasis*]	73/81	*caeA1*	67/75	*colA1b*	54/63	AMP ligase
11a	72	acyl carrier protein [*Micromonospora craniellae*]	66/80			*colA1a*	46/69	acyl carrier protein
12	417	DegT/DnrJ/EryC1/StrS familya aminotransferase family protein [*Streptoalloteichus tenebrarius*]	75/85	*caeP1*	70/80	*colP1*	62/74	aminotransferase
13	395	FAD-dependent oxidoreductase [*Amycolatopsis anabasis*]	76/84	*caeP2*	71/81	*colP2*	59/72	oxidase
14	2475	NRPS-T1PKS [*Streptoalloteichus tenebrarius*]	72/80	*caeA2*	68/77	*colA2*	58/69	NRPS/PKS
15	1058	non-ribosomal peptide synthetase [*Amycolatopsis anabasis*]	75/83	*caeA3*	71/79	*colA3*	52/65	NRPS
16	379	acyl-CoA dehydrogenase family protein [*Amycolatopsis anabasis*]	78/88	*caeB1*	79/87	*colB1*	62/74	acyl-CoA dehydrogenase
17	251	thioesterase II family protein [*Amycolatopsis anabasis*]	63/74	*caeA4*	62/72	*colA4*	48/63	thioesterase
18	145	response regulator transcription factor [*Amycolatopsis anabasis*]	68/76	*caeI1*	51/61	*colI1*	51/62	regulator
19	400	CrmL amidohydrolase [*Actinoalloteichus sp.* WH1-2216-6]	73/83	*caeD*	73/83	*colD*	61/73	amidohydrolase
20	351	SAM-dependent methyltransferase [*Streptomyces caatingaensis*]	65/74	*caeG1*	25/42	*colG1*	26/44	putative O-methyltransferase

21	550	class I adenylate-forming enzyme family protein [*Streptomyces caatingaensis*]	64/73	putative AMP-dependent synthetase/ligase
orf 1	394	MFS transporter [*Amycolatopsis sp.* NEAU-NG30]	89/91	
orf 2	227	FadR/GntR family transcriptional regulator [*Streptomyces caatingaensis*]	66/75	
orf 3	149	MerR family transcriptional regulator [*Amycolatopsis sp.* NEAU-NG30]	83/88	

[a] denotes amino acids
[b] denotes Identity/Similarity (%)

Table S4. Top FoldSeek structural search hits for putative hydroxylase Dip9 from PDB and AlphaFold Databases.

**Target**	**Database**	**Description**	**Scientific Name**	**Prob.**	**Seq. Id.**	**TM-score**	**Score**	**RMSD (Å)[a]**
4h2p-assembly1_A	PDB100	Tetrameric form of 2-methyl-3-hydroxypyridine-5-carboxylic acid oxygenase (MHPCO)	*Mesorhizobium japonicum MAFF 303099*	1	38.7	0.93	93	0.856
3alm-assembly2_B	PDB100	Crystal structure of 2-methyl-3-hydroxypyridine-5-carboxylic acid oxygenase, mutant C294A	*Mesorhizobium japonicum MAFF 303099*	1	38.8	0.93	93	0.888
3all-assembly1_A	PDB100	Crystal structure of 2-methyl-3-hydroxypyridine-5-carboxylic acid oxygenase, mutant Y270A	*Mesorhizobium japonicum MAFF 303099*	1	38.6	0.93	93	0.858
4h2n-assembly1_A	PDB100	Crystal structure of MHPCO, Y270F mutant	*Mesorhizobium japonicum MAFF 303099*	1	38.6	0.927	92	0.989
4bk2-assembly1_A-2	PDB100	Crystal structure of 3-hydroxybenzoate 6-hydroxylase uncovers lipid- assisted flavoprotein strategy for regioselective aromatic hydroxylation: Q301E mutant	*Rhodococcus jostii RHA1*	0.99	22.4	0.855	87	1.643
4bk3-assembly1_A-2	PDB100	Crystal structure of 3-hydroxybenzoate 6-hydroxylase uncovers lipid- assisted flavoprotein strategy for regioselective aromatic hydroxylation: Y105F mutant	*Rhodococcus jostii RHA1*	0.99	22.4	0.854	87	1.678
4bjz-assembly1_A	PDB100	Crystal structure of 3-hydroxybenzoate 6-hydroxylase uncovers lipid- assisted flavoprotein strategy for regioselective aromatic hydroxylation: Native data	*Rhodococcus jostii RHA1*	0.99	22.4	0.854	88	1.661
4bjy-assembly1_A-2	PDB100	Crystal structure of 3-hydroxybenzoate 6-hydroxylase uncovers lipid- assisted flavoprotein strategy for regioselective aromatic hydroxylation: Platinum derivative	*Rhodococcus jostii RHA1*	0.99	22.4	0.854	88	1.656

6sw1-assembly1_A	PDB100	Crystal Structure of P. aeruginosa PqsL: R41Y, I43R, G45R, C105G mutant	*Pseudomonas aeruginosa PAO1*	0.99	22.4	0.854	87	4.520
6sw2-assembly1_A	PDB100	Crystal Structure of P. aeruginosa PqsL in complex with 2-aminobenzoylacetate	*Pseudomonas aeruginosa PAO1*	0.99	20	0.850	84	4.130
AF-K0EM28-F1-model_v6	AFDB-Proteome	Monooxygenase	*Nocardia brasiliensis ATCC 700358*	0.99	25.4	0.874	87	1.283
AF-K0ETR9-F1-model_v6	AFDB-Proteome	FAD-binding monooxygenase	*Nocardia brasiliensis ATCC 700358*	0.99	22.4	0.868	86	1.388
AF-Q2FVW3-F1-model_v6	AFDB-Proteome	FAD-binding domain-containing protein	*Staphylococcus aureus subsp. aureus NCTC 8325*	0.99	21.3	0.868	87	2.790
AF-Q9HYR7-F1-model_v6	AFDB-Proteome	Probable FAD-dependent monooxygenase	*Pseudomonas aeruginosa PAO1*	0.99	19.6	0.862	88	1.687
AF-K0ETS7-F1-model_v6	AFDB-Proteome	2-polyprenyl-6-methoxyphenol hydroxylase-like oxidoreductase	*Nocardia brasiliensis ATCC 700358*	0.99	21.1	0.856	86	2.315
AF-A0A0E4AFG7-F1-model_v6	AFDB-SWISSPROT	Putative 2-heptyl-3-hydroxy-4(1H)-quinolone synthase AqdB1	*Rhodococcus erythropolis*	1	35.2	0.918	93	1.068
AF-A0A0E4AFH6-F1-model_v6	AFDB-SWISSPROT	Probable 2-heptyl-3-hydroxy-4(1H)-quinolone synthase AqdB2	*Rhodococcus erythropolis*	1	33.9	0.91	92	1.288
AF-B1MFK1-F1-model_v6	AFDB-SWISSPROT	2-heptyl-3-hydroxy-4(1H)-quinolone synthase	*Mycobacteroides abscessus ATCC 19977*	1	36.5	0.907	91	1.292
AF-O06489-F1-model_v6	AFDB-SWISSPROT	Putative FAD-dependent monooxygenase YetM	*Bacillus subtilis subsp. subtilis str. 168*	0.99	22.9	0.881	88	1.185
AF-H1ZZA4-F1-model_v6	AFDB-SWISSPROT	Aurachin C monooxygenase/isomerase	*Stigmatella aurantiaca*	0.99	22.1	0.869	88	1.544
AF-A0A3G8GK61-F1-model_v6	AFDB50	FAD-dependent monooxygenase	*Pigmentiphaga sp. H8*	1	31.8	0.895	91	1.063
AF-A0A1R4FIP6-F1-model_v6	AFDB51	Putative n-hydroxybenzoate hydroxylase	*Actinomycetales bacterium JB111*	1	26.9	0.889	90	1.083
AF-A0A177HPC6-F1-model_v6	AFDB52	FAD-dependent urate hydroxylase	*Streptomyces jeddahensis*	0.99	23.9	0.876	88	1.361
AF-A0A521KTI3-F1-model_v6	AFDB53	FAD-dependent monooxygenase	*Nitrospirota bacterium*	0.99	22.7	0.875	88	1.212
AF-A0A937BBR1-F1-model_v6	AFDB54	FAD-dependent monooxygenase	*Bacteroidia bacterium*	0.99	20	0.873	88	1.621

[a] RMSD values were calculated relative to the AlphaFold-predicted structure of Dip9.

Table S5. Top FoldSeek structural search hits for putative O-methyltransferase Dip20 from PDB and AlphaFold Databases.

Target	Database	Description	Scientific Name	Prob.	Seq. Id.	TM-score	Score	RMSD (Å)[a]
2r3s-assembly1_B	PDB100	crystal structure of a putative O-methyltransferase (Npun_R0239) from nostoc punctiforme pcc 73102 at 2.15 a resolution	*Nostoc punctiforme PCC 73102*	0.99	26.1	0.895	86	1.518
2r3s-assembly1_A	PDB100	crystal structure of a putative O-methyltransferase (npun_r0239) from nostoc punctiforme pcc 73102 at 2.15 a resolution	*Nostoc punctiforme PCC 73102*	0.99	26.1	0.893	86	1.471
7clf-assembly1_B	PDB100	PigF with SAH	*Serratia marcescens*	0.99	20.2	0.854	83	1.941
6c5b-assembly1_B	PDB100	Crystal Structure Analysis of LaPhzM	*Lysobacter antibioticus*	0.99	18.6	0.848	82	2.016
7clf-assembly1_A	PDB100	PigF with SAH	*Serratia marcescens*	0.99	21.4	0.846	82	2.353
3gwz-assembly1_A	PDB100	Structure of the Mitomycin 7-O-methyltransferase MmcR	*Streptomyces lavendulae*	0.99	22.4	0.84	82	2.661
4a6d-assembly1_A	PDB100	Crystal structure of human N-acetylserotonin methyltransferase (ASMT) in complex with SAM	*Homo sapiens*	0.99	22.3	0.839	83	2.094
8big-assembly1_A	PDB100	O-Methyltransferase Plu4895 in complex with SAH	*Photorhabdus laumondii subsp. laumondii TTO1*	0.98	14.1	0.827	78	3.331
5eeg-assembly1_A	PDB100	Crystal structure of carminomycin-4-O-methyltransferase DnrK in complex with tetrazole-SAH	*Streptomyces peucetius*	0.98	20	0.824	80	2.669
8bii-assembly4_E	PDB100	O-Methyltransferase Plu4895 (mutant H229N) in complex with SAH	*Photorhabdus laumondii subsp. laumondii TTO1*	0.98	13.8	0.824	78	3.318
AF-A0A8M3B5X1-F1-model_v6	AFDB-PROTEOME	Acetylserotonin O-methyltransferase	*Danio rerio*	0.99	20.4	0.842	83	2.139
AF-K0F7F0-F1-model_v6	AFDB-PROTEOME	O-methyltransferase	*Nocardia brasiliensis ATCC 700358*	0.99	21.3	0.84	84	2.431
AF-P46597-F1-model_v6	AFDB-PROTEOME	Acetylserotonin O-methyltransferase	*Homo sapiens*	0.99	22	0.84	83	2.056
AF-A3KNM1-F1-model_v6	AFDB-PROTEOME	Acetylserotonin O-methyltransferase	*Danio rerio*	0.99	20.8	0.831	82	2.286
AF-Q54B60-F1-model_v6	AFDB-PROTEOME	Probable inactive O-methyltransferase 11	*Dictyostelium discoideum*	0.98	14	0.831	80	2.387
AF-Q92056-F1-model_v6	AFDB-SWISSPROT	Acetylserotonin O-methyltransferase	*Gallus gallus*	0.99	21.1	0.85	84	2.039
AF-P10950-F1-model_v6	AFDB-SWISSPROT	Acetylserotonin O-methyltransferase	*Bos taurus*	0.99	21.4	0.846	83	2.115
AF-A0A172J1V3-F1-model_v6	AFDB-SWISSPROT	Phenazine O-methyltransferase PhzM	*Lysobacter antibioticus*	0.99	18.3	0.844	83	1.933
AF-Q8HZJ0-F1-model_v6	AFDB-SWISSPROT	Acetylserotonin O-methyltransferase	*Macaca mulatta*	0.99	21.4	0.842	83	2.231
AF-P46597-F1-model_v6	AFDB-SWISSPROT	Acetylserotonin O-methyltransferase	*Homo sapiens*	0.99	22	0.84	83	2.056
AF-A0A1F9RRF4-F1-model_v6	AFDB-50	O-methyltransferase domain-containing protein	*Elusimicrobia bacterium GWA2_69_24*	0.99	23.9	0.86	82	1.815
AF-W3RK91-F1-model_v6	AFDB-50	Hydroxyneurosporene-O-methyltransferase	*Afipia sp. P52-10*	0.99	19.1	0.853	83	1.861

AF-A0A1V2PPM1-F1-model_v6	AFDB-50	O-methyltransferase domain-containing protein	*Actinosynnema sp. ALI-1.44*	0.99	20.2	0.851	84	2.194
AF-A0A3C1EIH4-F1-model_v6	AFDB-50	SAM-dependent methyltransferase	*Deltaproteobacteria bacterium*	0.99	28.6	0.850	82	1.980
AF-M2Y0R8-F1-model_v6	AFDB-50	O-methyltransferase	*Galdieria sulphuraria*	0.99	21	0.849	83	2.275

[a] RMSD values were calculated relative to the AlphaFold-predicted structure of Dip20.

Table S6. Top FoldSeek structural search hits for putative amidotransferase Dip5 from PDB and AlphaFold Databases.

**Target**	**Database**	**Description**	**Scientific Name**	**Prob.**	**Seq. Id.**	**TM-score**	**Score**	**RMSD (Å)[a]**
7ylz-assembly2_B	PDB100	Unliganded form of hydroxyamidotransferase TsnB9	*Streptomyces sp. RM72*	1	37.5	0.938	91	0.703
7ylz-assembly1_A	PDB100	Unliganded form of hydroxyamidotransferase TsnB9	*Streptomyces sp. RM72*	1	37.1	0.934	91	0.673
1ct9-assembly2_C	PDB100	crystal structure of asparagine synthetase b from Escherichia coli	*Escherichia coli*	0.96	20.5	0.815	73	0.944
1ct9-assembly1_D	PDB100	crystal structure of asparagine synthetase b from Escherichia coli	*Escherichia coli*	0.96	20.7	0.814	73	0.966
1ct9-assembly1_A	PDB100	crystal structure of asparagine synthetase b from Escherichia coli	*Escherichia coli*	0.96	20.7	0.81	73	0.980
6gq3-assembly2_B	PDB100	human asparagine synthetase (AsnS) in complex with 6-diazo-5-oxo-l-norleucine (Don) at 1.85 a resolution	*Homo sapiens*	0.96	20.5	0.797	73	1.511
1mc1-assembly2_B	PDB100	beta-lactam synthetase with product (dgpc), amp and ppi	*Streptomyces clavuligerus*	0.91	16.1	0.757	68	1.864
1mbz-assembly2_B	PDB100	beta-lactam synthetase with trapped intermediate	*Streptomyces clavuligerus*	0.91	16.2	0.757	68	1.904
1mb9-assembly2_B	PDB100	beta-lactam synthetase complexed with atp	*Streptomyces clavuligerus*	0.91	16.2	0.755	68	2.044
1mbz-assembly1_A	PDB100	beta-lactam synthetase with trapped intermediate	*Streptomyces clavuligerus*	0.91	16.1	0.754	68	1.989
AF-Q9I231-F1-model_v6	AFDB-PROTEOME	asparagine synthase (glutamine-hydrolyzing)	*Pseudomonas aeruginosa PAO1*	1	44.9	0.97	97	0.565
AF-K0EYT1-F1-model_v6	AFDB-PROTEOME	asparagine synthase (glutamine-hydrolyzing)	*Nocardia brasiliensis ATCC 700358*	1	45.8	0.965	97	0.753
AF-Q9I781-F1-model_v6	AFDB-PROTEOME	asparagine synthase (glutamine-hydrolyzing)	*Pseudomonas aeruginosa PAO1*	1	40.8	0.961	96	0.649
AF-K0FD31-F1-model_v6	AFDB-PROTEOME	asparagine synthase (glutamine-hydrolyzing)	*Nocardia brasiliensis ATCC 700358*	1	43.3	0.959	96	0.766
AF-Q86A01-F1-model_v6	AFDB-PROTEOME	Asparagine synthetase	*Dictyostelium discoideum*	1	33.1	0.933	96	0.863
AF-O05272-F1-model_v6	AFDB-SWISSPROT	Asparagine synthetase [glutamine-hydrolyzing] 3	*Bacillus subtilis subsp. subtilis str. 168*	1	40.9	0.968	97	0.773
AF-P54420-F1-model_v6	AFDB-SWISSPROT	Asparagine synthetase [glutamine-hydrolyzing] 1	*Bacillus subtilis subsp. subtilis str. 168*	0.99	26	0.871	88	0.775
AF-P9WN32-F1-model_v6	AFDB-SWISSPROT	Putative asparagine synthetase [glutamine-hydrolyzing]	*Mycobacterium tuberculosis CDC1551*	0.99	24	0.859	88	0.978
AF-P64248-F1-model_v6	AFDB-SWISSPROT	Putative asparagine synthetase [glutamine-hydrolyzing]	*Mycobacterium tuberculosis variant bovis AF2122/97*	0.99	24	0.857	88	1.008
AF-P9WN33-F1-model_v6	AFDB-SWISSPROT	Putative asparagine synthetase [glutamine-hydrolyzing]	*Mycobacterium tuberculosis H37Rv*	0.99	24.2	0.857	88	1.007
AF-A0A1C5D2A1-F1-model_v6	AFDB-50	asparagine synthase (glutamine-hydrolyzing)	*Streptomyces sp. MnatMP-M17*	1	46.2	0.97	97	0.847

AF-A0A3R8QEW7-F1-model_v6	AFDB-50	asparagine synthase (glutamine-hydrolyzing)	*Streptomyces griseofuscus*	1	48.4	0.97	97	0.768
AF-A0A2T7M9Z8-F1-model_v6	AFDB-50	asparagine synthase (glutamine-hydrolyzing)	*Streptomyces sp. CS014*	1	46.3	0.969	97	0.794
AF-A0A6G2PY81-F1-model_v6	AFDB-50	asparagine synthase (glutamine-hydrolyzing)	*Streptomyces sp. SID685*	1	47.9	0.968	97	0.728

[a] RMSD values were calculated relative to the AlphaFold-predicted structure of Dip5.

Table S7. Top FoldSeek structural search hits for putative synthetase/ligase Dip21 from PDB and AlphaFold Databases.

**Target**	**Database**	**Description**	**Scientific Name**	**Prob.**	**Seq. Id.**	**TM-score**	**Score**
1mdf-assembly1_A	PDB100	crystal structure of DhbE in absence of substrate	*Bacillus subtilis*	1	28.3	0.916	90
7tz4-assembly1_A	PDB100	Salicylate Adenylate PchD from Pseudomonas aeruginosa containing 4-cyanosalicyl-AMS	*Pseudomonas aeruginosa*	0.99	28.8	0.903	88
7tyb-assembly1_A	PDB100	Salicylate Adenylate PchD from Pseudomonas aeruginosa containing salicyl-AMS	*Pseudomonas aeruginosa PAO1*	0.99	28.8	0.898	87
7kyd-assembly1_A	PDB100	Drosophila melanogaster long-chain fatty-acyl-CoA synthetase CG6178	*Drosophila melanogaster*	0.99	20.8	0.87	85
4wv3-assembly2_B	PDB100	Crystal structure of the anthranilate CoA ligase AuaEII in complex with anthranoyl-AMP	*Stigmatella aurantiaca*	0.99	20.9	0.867	84
2v7b-assembly2_B	PDB100	Crystal structures of a benzoate CoA ligase from Burkholderia xenovorans LB400	*Paraburkholderia xenovorans LB400*	0.99	20	0.867	81
4gxr-assembly1_A	PDB100	Structure of ATP bound RpMatB-BxBclM chimera B3	*Rhodopseudomonas palustris CGA009*	0.99	21.6	0.866	82
4gxq-assembly3_C	PDB100	Crystal Structure of ATP bound RpMatB-BxBclM chimera B1	*Rhodopseudomonas palustris CGA009*	0.99	22.3	0.863	82
4wv3-assembly1_A	PDB100	Crystal structure of the anthranilate CoA ligase AuaEII in complex with anthranoyl-AMP	*Stigmatella aurantiaca*	0.99	21.3	0.862	83
5bsw-assembly2_B	PDB100	Crystal structure of 4-coumarate:CoA ligase delta-V341 mutant complexed with feruloyl adenylate	*Nicotiana tabacum*	0.99	21.1	0.86	84
AF-K0EMM1-F1-model_v6	AFDB-PROTEOME	Hydroxybenzoate-AMP ligase	*Nocardia brasiliensis ATCC 700358*	1	28.6	0.924	91
AF-K0ER74-F1-model_v6	AFDB-PROTEOME	(2,3-dihydroxybenzoyl)adenylate synthase	*Nocardia brasiliensis ATCC 700358*	1	27.9	0.897	89
AF-K0F5E8-F1-model_v6	AFDB-PROTEOME	2,3-dihydroxybenzoate-AMP ligase	*Nocardia brasiliensis ATCC 700358*	0.99	28	0.884	85
AF-I6Y0X0-F1-model_v6	AFDB-PROTEOME	Long-chain-fatty-acid--CoA ligase FadD13	*Mycobacterium tuberculosis H37Rv*	0.99	28.2	0.879	87
AF-A0A8R1XRS3-F1-model_v6	AFDB-PROTEOME	Uncharacterized protein	*Onchocerca volvulus*	0.99	21.2	0.874	86
AF-P40871-F1-model_v6	AFDB-SWISSPROT	2,3-dihydroxybenzoate-AMP ligase	*Bacillus subtilis subsp. subtilis str. 168*	1	28.5	0.912	90
AF-P80436-F1-model_v6	AFDB-SWISSPROT	Triostin synthetase I	*Streptomyces triostinicus*	0.99	29.6	0.905	88
AF-Q84HC5-F1-model_v6	AFDB-SWISSPROT	2-hydroxy-7-methoxy-5-methyl-1-naphthoate--CoA ligase	*Streptomyces carzinostaticus*	1	27.5	0.902	90
AF-B7N7R2-F1-model_v6	AFDB-SWISSPROT	Crotonobetaine/carnitine--CoA ligase	*Escherichia coli UMN026*	0.99	20.2	0.888	86
AF-Q7X279-F1-model_v6	AFDB-SWISSPROT	Salicylyl-CoA synthase / salicylate adenylyltransferase	*Streptomyces sp.*	0.99	26	0.886	88
AF-A0A1I1I0A3-F1-model_v6	AFDB-50	Cyclohexanecarboxylate-CoA ligase	*Tropicimonas isoalkanivorans*	1	25.6	0.905	89

AF-A0A2M8QKD6-F1-model_v6	AFDB-50	AMP-dependent synthetase	*Sphingobium sp. LB126*	0.99	23.6	0.882	86
AF-A0A2A4V3N2-F1-model_v6	AFDB-50	Uncharacterized protein	*Alphaproteobacteria bacterium*	0.99	23.9	0.88	86
AF-A0A9E1SR13-F1-model_v6	AFDB-50	3-methylmercaptopropionyl-CoA ligase	*Rhodospirillaceae bacterium*	0.99	22.8	0.879	84
AF-A0A4R7I3P8-F1-model_v6	AFDB-50	Acyl-CoA synthetase (AMP-forming)/AMP-acid ligase II	*Ilumatobacter fluminis*	0.99	23.9	0.877	84

Table S8. Top FoldSeek structural search hits for putative hydrolase Dip10 from PDB and AlphaFold Databases.

**Target**	**Database**	**Description**	**Scientific Name**	**Prob.**	**Seq. Id.**	**TM-score**	**Score**
4l0c-assembly6_F	PDB100	Crystal structure of the N-Fopmylmaleamic acid deformylase Nfo(S94A) from Pseudomonas putida S16	*Pseudomonas putida S16*	0.99	19.8	0.818	85
4l0c-assembly7_G	PDB100	Crystal structure of the N-Fopmylmaleamic acid deformylase Nfo(S94A) from Pseudomonas putida S16	*Pseudomonas putida S16*	0.99	20	0.81	84
3kxp-assembly1_A	PDB100	Crystal Structure of E-2-(Acetamidomethylene)succinate Hydrolase	*Mesorhizobium loti*	0.99	19.6	0.806	86
1zoi-assembly1_B	PDB100	Crystal Structure of a Stereoselective Esterase from Pseudomonas putida IFO12996	*Pseudomonas putida*	0.99	15.7	0.791	85
1a88-assembly1_C	PDB100	CHLOROPEROXIDASE L	*Streptomyces lividans*	0.99	16.5	0.79	85
5h3h-assembly1_A	PDB100	Esterase (EaEST) from Exiguobacterium antarcticum	*Exiguobacterium antarcticum B7*	0.99	13.5	0.788	84
1a8q-assembly1_A	PDB100	BROMOPEROXIDASE A1	*Kitasatospora aureofaciens*	0.99	17.6	0.788	84
3ia2-assembly2_D	PDB100	Pseudomonas fluorescens esterase complexed to the R-enantiomer of a sulfonate transition state analog	*Pseudomonas fluorescens*	0.99	14.4	0.787	84
3hea-assembly1_A	PDB100	The L29P/L124I mutation of Pseudomonas fluorescens esterase	*Pseudomonas fluorescens*	0.99	14.4	0.787	84
3hi4-assembly2_D	PDB100	Switching catalysis from hydrolysis to perhydrolysis in P. fluorescens esterase	*Pseudomonas fluorescens*	0.99	14	0.787	84
AF-K0F1F1-F1-model_v6	AFDB-PROTEOME	Hydrolase	*Nocardia brasiliensis ATCC 700358*	0.99	15.9	0.797	83
AF-K0ELX9-F1-model_v6	AFDB-PROTEOME	Hydrolase	*Nocardia brasiliensis ATCC 700358*	0.99	19.4	0.794	81
AF-Q9I0C5-F1-model_v6	AFDB-PROTEOME	Chloroperoxidase	*Pseudomonas aeruginosa PAO1*	0.99	17.2	0.788	85
AF-A0A0H3GV49-F1-model_v6	AFDB-PROTEOME	Non-heme chloroperoxidase	*Klebsiella pneumoniae subsp. pneumoniae HS11286*	0.99	17.1	0.788	85
AF-Q8ZLI9-F1-model_v6	AFDB-PROTEOME	Pimeloyl-[acyl-carrier protein] methyl ester esterase	*Salmonella enterica subsp. enterica serovar Typhimurium str. LT2*	0.99	18.9	0.788	82
AF-Q88FY3-F1-model_v6	AFDB-SWISSPROT	N-formylmaleamate deformylase	*Pseudomonas putida KT2440*	0.99	17.3	0.812	86
AF-Q15N09-F1-model_v6	AFDB-SWISSPROT	Pimeloyl-[acyl-carrier protein] methyl ester esterase	*Paraglaciecola sp. T6c*	0.99	18.1	0.803	84
AF-Q609V0-F1-model_v6	AFDB-SWISSPROT	Pimeloyl-[acyl-carrier protein] methyl ester esterase	*Methylococcus capsulatus str. Bath*	0.99	17.2	0.802	83
AF-O32234-F1-model_v6	AFDB-SWISSPROT	AB hydrolase superfamily protein YvaM	*Bacillus subtilis subsp. subtilis str. 168*	0.99	13.6	0.796	83
AF-B5FFE9-F1-model_v6	AFDB-SWISSPROT	Pimeloyl-[acyl-carrier protein] methyl ester esterase	*Aliivibrio fischeri MJ11*	0.99	14.2	0.795	83

AF-A0A7W3NKJ3-F1-model_v6	AFDB-50	Pimeloyl-ACP methyl ester carboxylesterase	*Streptomyces murinus*	1	39.3	0.948	95
AF-A0A8J3J234-F1-model_v6	AFDB-50	AB hydrolase-1 domain-containing protein	*Actinocatenispora rupis*	1	53.8	0.947	96
AF-A0AAU2UQC5-F1-model_v6	AFDB-50	Alpha/beta fold hydrolase	*Streptomyces sp. NBC_00012*	1	40.5	0.945	94
AF-A0A426R654-F1-model_v6	AFDB-50	Alpha/beta fold hydrolase	*Rhodococcus sp. Eu-32*	1	46.9	0.945	94
AF-M2QRQ5-F1-model_v6	AFDB-50	Putative hydrolase	*Amycolatopsis azurea DSM 43854*	1	96.2	0.94	90

Table S9. Antibacterial activities of dipyrimicin A and B reported as minimum inhibitory activity.

	Antibacterial activity (μg/mL)		
	Dipyrimicin A **(1)**	Dipyrimicin B **(2)**	Tetracycline (control)
Bacillus spizizenii ATCC 6633	32	>128	0.25
Escherichia coli MG1655 ATCC 700926	128	>128	2
Pseudomonas aeruginosa ATCC 27853	>128	NT[a]	8
Staphylococcus aureus ATCC 25923	64	NT[a]	0.25

[a]NT = not tested.

Table S10. ML-predicted antibacterial activity probability of BGCs encoding 2,2′-BP genes described in **Figure 4**.

**Strain**	**NCBI RefSeq_Assembly**	***cae* % similarity[a]**	***col* % similarity[a]**	**Average antibacterial activity score**
Actinosynnema sp. ALI-1.44	GCF_001984155.1_ASM198415v1_genomic	52%	59%	74%
Streptomyces caatingaensis CMAA 1322	GCF_001187435.1_ASM118743v1_genomic	48%	38%	74%
Amycolatopsis anabasis EGI 650086	GCF_009765355.1_ASM976535v1_genomic	80%	59%	74%
Nocardia panacis YIM PH 21724	GCF_003598715.1_ASM359871v1_genomic	72%	55%	73%
Kitasatospora albolonga YIM 101047	GCF_002082585.1_ASM208258v1_genomic	68%	88%	70%
Streptomyces sp. SID14515	GCF_010548505.1_ASM1054850v1_genomic	60%	66%	67%
Streptomyces sp. Ncost-T6T-1	GCF_002705685.1_ASM270568v1_genomic	60%	77%	63%
Streptomyces filamentosus NRRL 15998	GCF_000156455.1_ASM15645v1_genomic	56%	70%	60%
Micromonospora craniellae LHW63014	GCF_014764405.1_ASM1476440v1_genomic	60%	62%	59%
Streptomyces filamentosus NRRL 11379	GCF_000156695.2_ASM15669v2_genomic	60%	74%	59%
Streptomyces sp. Cmuel-A718b	GCF_900092005.1_IMG-taxon_2657245729_annotated_assembly_genomic	60%	74%	56%
Micromonospora tulbaghiae CNY-010	GCF_003612775.1_ASM361277v1_genomic	56%	59%	54%
Streptomyces sp. CS149	GCF_003024215.1_ASM302421v1_genomic	60%	70%	54%
Streptomyces sp. CAI 127	GCF_013363655.1_ASM1336365v1_genomic	60%	74%	52%
Streptomyces sp. TSRI0261	GCF_001905485.1_ASM190548v1_genomic	60%	74%	51%
Streptomyces sp. CB00271	GCF_014495705.1_ASM1449570v1_genomic	60%	74%	50%

Streptomyces sp. WA6-1-16	GCF_020341595.1_ASM2034159v1_genomic	60%	74%	49%
Streptomyces sp. CB02613	GCF_002803175.1_ASM280317v1_genomic	60%	74%	48%

[a]from AntiSMASH 5 KnownClusterBlast

References

(1) Suemori, A. Conserved and Non-Conserved Residues and Their Role in the Structure and Function of p-Hydroxybenzoate Hydroxylase. *Protein Eng. Des. Sel.* **2013**, *26* (7), 479–488. https://doi.org/10.1093/protein/gzt026.
